# High prevalence of plasmid-mediated Fosfomycin resistance in waterfowl-derived *Escherichia coli* strains: insights into genetic context and transmission dynamics in China

**DOI:** 10.3389/fvets.2025.1481822

**Published:** 2025-03-21

**Authors:** Shaqiu Zhang, Jing Yang, Qian Yang, Qianlong Li, Zhijun Zhong, Mingshu Wang, Renyong Jia, Shun Chen, Mafeng Liu, Dekang Zhu, Xinxin Zhao, Ying Wu, Qiao Yang, Juan Huang, Xumin Ou, Di Sun, Bin Tian, Zhen Wu, Yu He, Anchun Cheng

**Affiliations:** ^1^Avian Disease Research Center, College of Veterinary Medicine, Sichuan Agricultural University, Chengdu, China; ^2^Institute of Veterinary Medicine and Immunology, Sichuan Agricultural University, Chengdu, China; ^3^Key Laboratory of Animal Disease and Human Health of Sichuan Province, Sichuan Agricultural University, Chengdu, China; ^4^Engineering Research Center of Southwest Animal Disease Prevention and Control Technology, Ministry of Education, Chengdu, China

**Keywords:** waterfowl, *Escherichia coli*, Fosfomycin resistance, *fos*A3, plasmid -mediated

## Abstract

Fosfomycin (FOS) is a critical antibiotic for treating multi-drug resistant (MDR) *Enterobacteriaceae* infections, but its effectiveness is jeopardized by the dissemination of plasmids encoding enzymes that modify FOS. Despite the prohibition on its use in animal breeding in China, 100 strains of *Escherichia coli* (*E. coli*) exhibiting high resistance to FOS (MIC≥512 mg/L) were isolated from samples of waterfowl origin collected in Hainan, Sichuan, and Anhui. These strains commonly carried the *fos*A*3* (88/100, 88.0%). In addition, 21 other antimicrobial resistance genes (ARGs) were detected in these strains, with high positivity rates for *tet*A, *aphA1*, *sul2*, *folR*, *qnrS*, and *bla*_CTX-M_. It is noteworthy that there was a significant positive correlation between the *fos*A3 and *bla*_CTX-M_ (OR = 15.162, 95% CI: 1.875–122.635). The results of pulsed-field gel electrophoresis (PFGE) demonstrated the existence of multiple dispersed clonal clusters. Multilocus sequence typing (MLST) analysis identified 45 ST types, with ST48 and ST10 representing the most dominant clones. In the conjugation experiments, 53 *fos*A-like genes positive transconjugants were obtained with measurable conjugation frequency, which strongly demonstrated that these *fos*A3 may mainly locate on different types of plasmids possessing an efficient transmission ability. Whole genome sequencing (WGS) analysis further showed that the *fos*A3 was co-localized with the *bla*_CTX-M_ on plasmids that showed a high degree of similarity in genetic structure. Of particular interest is the observation that the *fos*A3 is frequently accompanied by IS*26* on either side of the gene. This structure may play a pivotal role in the horizontal transfer of the *fos*A3. The study revealed the alarming prevalence of FOS resistance in *E. coli* of waterfowl origin and delved deeply into the genetic characteristics and transmission mechanisms of the *fos*A3. The discovery of plasmid-mediated, transmissible FOS resistance in waterfowl *E. coli* poses a threat to “One Health”. There’s an urgent need for thorough monitoring and control measures against FOS resistance.

## Introduction

Antimicrobial resistance (AMR) represents a significant challenge to global public health (1). It is projected that by 2050, AMR will be responsible for the deaths of approximately 10 million individuals (2). In contrast to the rapid spread and development of AMR, the development of novel antimicrobial has been relatively sluggish (3). Consequently, the scientific community has initiated a re-evaluation of the potential benefits of older antimicrobial. Fosfomycin (FOS), an antimicrobial agent first discovered in the 1960s, has garnered renewed interest in recent years (4). FOS is bactericidal against a variety of multi-drug resistant bacteria (5). For example, carbapenem-resistant *Enterobacteriaceae* (CRE), extended-spectrum *β*-lactamase producing *Enterobacteriaceae* (ESBL-PE), methicillin-resistant *Staphylococcus aureus*, and vancomycin-resistant *Enterococcus faecalis*, all of which are classified as priority pathogens by the World Health Organization (6). However, with the increasing use of FOS, there has also been a rise in instances of FOS-resistant pathogens, particularly those harboring plasmid-encoded FOS resistance modifier genes (7).

Previous studies have demonstrated that two primary mechanisms mediate resistance to FOS in *Enterobacteriaceae*: (i) Chromosomal mutations in the FOS target gene *murA* or in FOS uptake genes (e.g., *uhpT*, *uhpA*, *glpT*, *cyaA* and *ptsI*); and (ii) Plasmid encoded inactivating enzymes (including FosA variants, FosB, FosC, FosX, FosK, FosD, FosE, FosI, FosL, FomA and FomB). Although the use of FOS in animals has long been prohibited in China, there has been a notable increase in the incidence of FOS resistance among bacteria of animal origin (8). In addition, recent studies indicate that plasmid-mediated *fos*A3 is the predominant mechanism conferring resistance to FOS within *Enterobacteriaceae* in China (9). The presence of bacteria harboring the *fos*A3 poses a significant risk to both animal and human health under the “One Health” concept. Notably, The *fos*A3 has been identified in *Enterobacteriaceae* isolated from a diverse array of animal sources in China, including pigeons, pigs, chickens, companion animals, cattle, and rodents (10, 11). It has been observed that livestock and poultry populations within China have become reservoirs for FOS-resistant bacteria. Waterfowl excrement serves as a reservoir for novel ARGs, with various ARGs having been identified in the feces of waterfowl (12–16). The rapid dissemination of *fos*A3 is evidently associated with IncF plasmid and IS*26* (9, 17). Plasmids carrying the *fos* gene are frequently found to co-occur with other ARGs, thereby increasing the likelihood that FOS resistance will be selected concurrently alongside these additional resistance mechanisms. Previous studies have shown that coexistence between *fos*A3 and other ARGs is mediated by IS*26*-type complex transposons (18).

Our research team has a long-standing interest in the study of bacterial resistance in waterfowl, and has produced a series of findings, particularly in the area of MDR (15, 16, 19). Among our findings, we have made significant strides in understanding the global distribution of *E. coli* harboring the *mcr-1*, and have conducted a comprehensive genomic analysis to elucidate the spread of *mcr-1* and its broader public health implications (13, 20). In the field of tigecycline resistance, our initial research elucidated the molecular propagation of *tet*(X4) in the avian environment of Sichuan Province (15, 21). In the area of carbapenem resistance, we have identified for the first time *E. coli* carrying the *bla*_NDM-1_ in waterfowl-origin bacteria from a tropical island in China, a finding that underscores the urgency of tightening control of the spread of *bla*_NDM-1_-producing *E. coli* in the region (22).

The prevalence of drug resistance in waterfowl is increasing on a global scale. The spread of AMR microorganisms and associated genes is not geographically restricted and can easily cross different regions and hosts. It is therefore imperative that global cooperation should be established to address this challenge, through the development of policies, the implementation of preventive measures, and the involvement of all relevant parties in the entire chain.

Despite some advances in research on FOS resistance, our knowledge of FOS resistance in *E. coli* in waterfowl remains limited. FOS, a long-established antimicrobial agent, has recently been the subject of renewed interest from the research community due to its demonstrated bactericidal activity against a wide range of antibiotic-resistant bacteria (ARB). However, with the increased utilization of FOS, the prevalence of ARB harboring plasmid-encoded FOS resistance related genes has also increased. Accordingly, the objective of this study was to conduct a comprehensive investigation into the resistance characteristics of *E. coli* derived from waterfowl to FOS, and to analyze the genetic characteristics of the *fos*A3 and its transmission risk. This is crucial for enhancing our comprehension of and ability to control ARB in waterfowl.

## Materials and methods

### Screening of FOS-resistant *Escherichia coli* from previously collected strains

Our research subjects are strains of *E. coli* isolated and preserved in the laboratory from 2019 to 2023, which all originate from representative waterfowl species in China, including Sichuan mallard ducks, Hainan Jiaji ducks, and Anhui white geese (16, 23, 24). The sample collection sites cover the main breeding areas of these waterfowl, ensuring the geographical representativeness of the samples. Specifically, the sources of the samples include duck intestinal contents or pericardial effusion, cloacal swabs and fresh feces, all of which were collected onsite at breeding farms. During the collection process, we had detailed exchanges with local veterinarians and learned that these breeding farms have a history of using antibiotics.

The authenticated *E. coli* strains were meticulously stored in 20% glycerol at −80°C. Subsequently, these isolates were cultured on MacConkey agar plates supplemented with 128 mg/L of FOS and 25 mg/L of glucose-6-phosphate (G-6-P), with the aim of identifying FOS-resistant *E. coli* strains among our previously collected strains.

### DNA extraction

Total genomic DNA was extracted from bacterial cultures using the TIANamp Bacteria DNA Kit (TianGen Biotech, Beijing, China, #DP302) according to the manufacturer’s instructions, and the DNA quality was measured by UV absorbance (ND1000, Nanodrop, Thermo Fisher Scientific). The DNA samples were stored at −20°C.

### Identification of *fosA*-like genes and detection of other ARGs

In order to determine which types of *fos*A-like genes are carried by FOS -resistant *E. coli*, 10 previously reported *fos*A-like genes (*fos*A to *fos*A10) were identified through PCR. Furthermore, these strains were tested for the presence of other ARGs using primers that had previously been employed for this purpose. All primers are shown in Supplementary Table S1. PCR experiments were performed in a 20 μL volume containing 2 μL of template DNA, 10 μL of 2 × Hieff^®^ Robust PCRMaster Mix (With Dye) (Yeasen Biotechnology, Shanghai, China), 6 μL of ddH_2_O, and 1 μL of each primer. The PCR amplification program was set according to the instructions of the amplification enzyme. PCR products were separated by gel electrophoresis in a 1.0% agarose gel stained with GoldView™ (Sangon Biotech, Shanghai, China), visualized under UV light and photographed with a gel documentation system (Bio Rad, Hercules, United States). The positive PCR products were sent to Tsingke Biotechnology Co., Ltd. for DNA sequencing. Finally, the obtained sequences were analyzed by using BLAST tools of online gene database.^1^

### Antimicrobial susceptibility testing

FOS-resistant strains were subjected to susceptibility testing for 15 antibacterial agents according to Clinical & Laboratory Standards Institute (CLSI) standards, employing micro broth dilution and agar dilution methodologies. The following antibacterial agents were included in the study: imipenem (IPM, #S24020), tigecycline (TGC, #MB1246), polymyxin B (PB, #MB1188), enrofloxacin (ENR, #MB1359-1), ofloxacin (OFX, #MB1659-1) and fosfomycin (FOS, #MB1110) were procured from Dalian Meilun Biotechnology (Dalian, China). Ampicillin (AMP, #A830931), cefotaxime (CTX, #C804340), florfenicol (FLM, #F809685), nitrofurantoin (NIT, #N814882), amikacin (AMK, #A837351) were obtained from Shanghai Macklin Biochemical Technology (Shanghai, China). Additionally, tetracycline (TET, #B25579), sulfamethoxazole (SMZ, #B24157), gentamicin (GEN, #V30125) and ceftazidime (CAZ, #S17139) were also purchased from Shanghai yuanye Bio-Technology (Shanghai, China). In order to idenitfy the MIC values of FOS, the agar dilution assay must be performed on Mueller-Hinton agar, which has been supplemented with 25 mg/L G-6-P. The MIC breakpoints were determined according to CLSI criteria (25). For TGC, breakpoints were referenced against the European Committee on Antimicrobial Susceptibility Testing (EUCAST) criteria, while those for ENR were based on a previous study (26).

### Pulsed field gel electrophoresis

X*baI*-PFGE was conducted using X*baI* endonuclease in accordance with the previously described methodology (22). In summary, Genomic DNA of *E. coli* was prepared using the restriction enzyme *X*baI (TaKaRa, Japan) and electrophoresed in SeaKem Gold Agarose (Lonza, Swiss). DNA fragments were separated by PFGE on a CHEF-Mapper XA PFGE system (Bio-Rad, United States) with a pulse angle of 120° (pulse times 6.76–35.38 s) for 18 h at 6 V/cm and 14°C. After electrophoresis, the gel was stained with SuperRed/GelRed nucleic acid stain (Biosharp, China) and imaged using a gel imaging system. Subsequent to this, phylogenetic analyses of the PFGE patterns were performed utilizing Gel Jv 2.0 software, and clustering analyses were conducted using the unweighted group average method (UPGMA).

### Phylogenetic typing and MLST detection

A classification of *E. coli* into six types, A, B1, B2, C, D, and E, as well as two branches, clade I and clade II, was established by employing a quadruple PCR and two individual PCRs, in accordance with the method initially proposed (27). In accordance with the guidelines set forth by the MLST database^2^ as proposed (28), 7 housekeeping genes (*adk*, *fumC*, *gyrB*, *icdF*, *mdh*, *purE*, *recA*) were subjected to PCR amplification for MLST analysis. All primers are shown in Supplementary Table S1.

### Conjugation experiments

Conjugation experiments were conducted using the liquid ligation method with *E. coli* J53 as the recipient bacterium. Specifically, *E. coli* J53 was co-cultured with *fos*A-like gene-positive *E. coli*, after which the mixture was centrifuged and processed. The resulting solution was then spread on agar plates containing specific components, including 256 mg/L FOS, 150 mg/L NaN_3_, and 25 mg/L G-6-P. Subsequently, the colonies were picked from the plates and subjected to PCR identification to detect the presence of the *fos*A-like gene. The positive strain was retained as a transconjugant. In addition, the transconjugants were tested for their MIC against antimicrobial agents, and the ARGs carried by these transconjugants were analyzed. Furthermore, the conjugation frequency was determined in accordance with the previous study (14). Briefly, donor and recipient bacteria were activated and diluted to OD600 = 0.5, mixed 1:1 and incubated at 37°C for 24 h, and then diluted with saline to appropriate concentrations. Hundred microliter of the mixture was spread on LB agar containing 256 mg/L FOS and 150 mg/L NaN_3_, and 100 μL of *E. coli* J53 was spread on LB agar containing 150 mg/L NaN_3_, both incubated at 37°C for 24 h. Individual colonies on the FOS + NaN_3_ LB agar were the transconjugant (note as T), and those on the NaN_3_ LB agar were the receptor (note as T). On NaN_3_ LB agar were the acceptors (note as R), and the frequency of conjugation was calculated as T/R. The experiment was repeated three times.

### Plasmid replicon typing

The PCR-based replicon typing (PBRT) technique was employed for the detection of plasmid incompatibility groups in transconjugants as well as their donor bacteria, as previously outlined (19). All primers are shown in Supplementary Table S1.

### Whole-genome sequencing and analysis

In this study, 3 strains were selected for genome sequencing: HN68, carrying the *fos*A3 + *bla*_CTX-M-55_; LA3, carrying the *fos*A3 + *bla*_NDM-5_; and BHD6, carrying the *fos*A3 + *mcr-1.1*. We commissioned Paysono Bioscience and Technology Co., Ltd. to perform the precise sequencing work using Illumina NovaSeq and Oxford Nanopore ONT sequencing platforms. The process of annotating bacterial genomes utilizing the NCBI prokaryotic genome annotation pipeline (29). Additionally, an in-depth analysis of ARGs, MGEs, and serotypes was conducted using the Centre for Genomic Epidemiology.^3^ In order to visualize the genomic structure and differences between plasmids, CGview^4^ was employed to map the plasmid comparative genomic circles. Finally, a linear comparative genome map was plotted using the Easyfig soft.

### Data availability

We have submitted the genomic data of three *fos*A3 positive strains to the NCBI database. The strains are HN68, LA3, and BHD6, and the corresponding BioProject IDs are PRJNA1057772, PRJNA1057764, and PRJNA1057743 respectively, which also including plasmids information.

### Analysis and processing of data

Based on the isolation results of the FOS-resistant bacteria, the data of the antimicrobial sensitivity test and the detection results of the ARGs, the associations between antimicrobial resistance and the ARGs, and within the ARGs between different regions were statistically analyzed by using the *χ*^2^-test in the SPSS (Version 27.0) software. These differences were considered statistically significant when *p* < 0.05. To further quantify this association, it was calculated by odds ratio (OR) and 95% confidence interval (95% CI). In this case, an OR value of less than 1 indicates a negative correlation, whereas an OR value of greater than 1 indicates a positive correlation. To analyze the results of plasmid conjugation transfer frequency, one-way ANOVA was performed using GraphPad Prism 8 software. These results were considered statistically significant when *p* < 0.05.

## Results

### Isolation of FOS-resistant strains and detection of the *fos*A-like gene

A total of 100 FOS-resistant *E. coli* isolates were obtained from waterfowl isolates obtained from Hainan, Sichuan, and Anhui. The results showed that the isolation rate of resistant strains in Sichuan was significantly higher than that in Hainan and Anhui (*p* = 0.047), while no significant difference was observed between the latter two regions (Table 1).

**Table 1:** Isolation of fosfomycin-resistant *E. coli*.

	Hainan	Anhui	Sichuan	*P*-value
Number	31/311	30/284	39/238	
Rate of resistance	9.97%a	10.56%a	16.39%b	0.047

Each subscript letter indicates that at the 0.05 level, the column proportions for these categories are not significantly different from each other.

In this study, we identified 10 types of *fos*A-like genes (*fos*A-*fos*A10) in 100 strains of FOS-resistant bacteria. The results revealed that the prevalence of *fos*A3 was the highest at 88.0%, followed by *fos*A (11.0%), *fos*A7 (3.0%), *fos*A10 (3.0%), and *fos*A6 (2.0%). It is noteworthy that all resistant strains carried the *fos*A-like genes, while the presence of *fos*A2, *fos*A4, *fos*A5, *fos*A8, and *fos*A9 was not observed in this study (Supplementary Table S2).

### Antimicrobial resistance in FOS-resistant isolates

**Figure 1:**
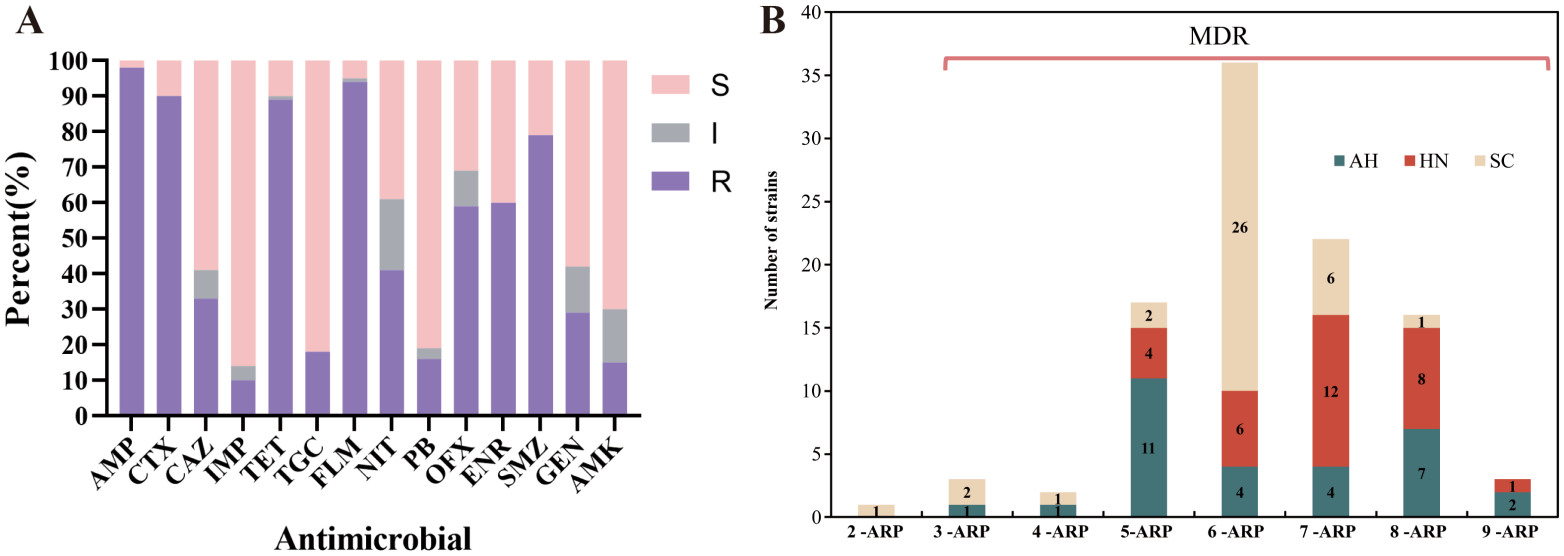
Antimicrobial resistance rate and MDR classification of FOS resistant strains. In panel **(A)**, the histogram with different colors represents the percentages of sensitivity (s), intermediation (I) and resistance (R), respectively. In panel **(B)**, the histogram shows the number of strains with different ARP.

The results revealed that all strains exhibited high levels of resistance to FOS (MIC≥512 mg/L). Among the remaining 14 antimicrobial agents, AMP, FLM, CTX, and TET demonstrated significantly higher resistance rates of up to 98.0, 94.0, 90.0, and 89.0%, respectively. SMZ, ENR, OFX, and NIT also displayed relatively high resistance rates of 79.0, 60.0, 59.0, and 41.0%, respectively. Additionally, CAZ, GEN, TGC, PB, and AMK showed resistance rates of 33.0, 29.0, 18.0, 16.0, and 15.0% respectively; whereas IMP exhibited the lowest resistance rate at 10.0% (Figure 1A). Strains positive for the *fos*A demonstrated variable resistance to different types of antimicrobials with a staggering 99.0% exhibiting multi-drug resistance. The antimicrobial-resistant-patterns (ARP) ranged from 3 to 9 indicating the breadth and severity of the resistance (Figure 1B). The only exception was a strain that was solely resistant to SMZ and FOS. Among the multi-resistant bacteria, a large proportion were resistant to antimicrobial drugs belonging to classes 5 through 8, especially classes 6 and 7 reaching 36.0 and 22.0%, respectively. These *fos*A positive strains displayed various phenotypic patterns with a total of 69 resistance profiles detected. Of these, the most common pattern was the 6-ARP, which encompassed 36 strains in total (Figure 1B).

### Analysis of ARGs

**Figure 2:**
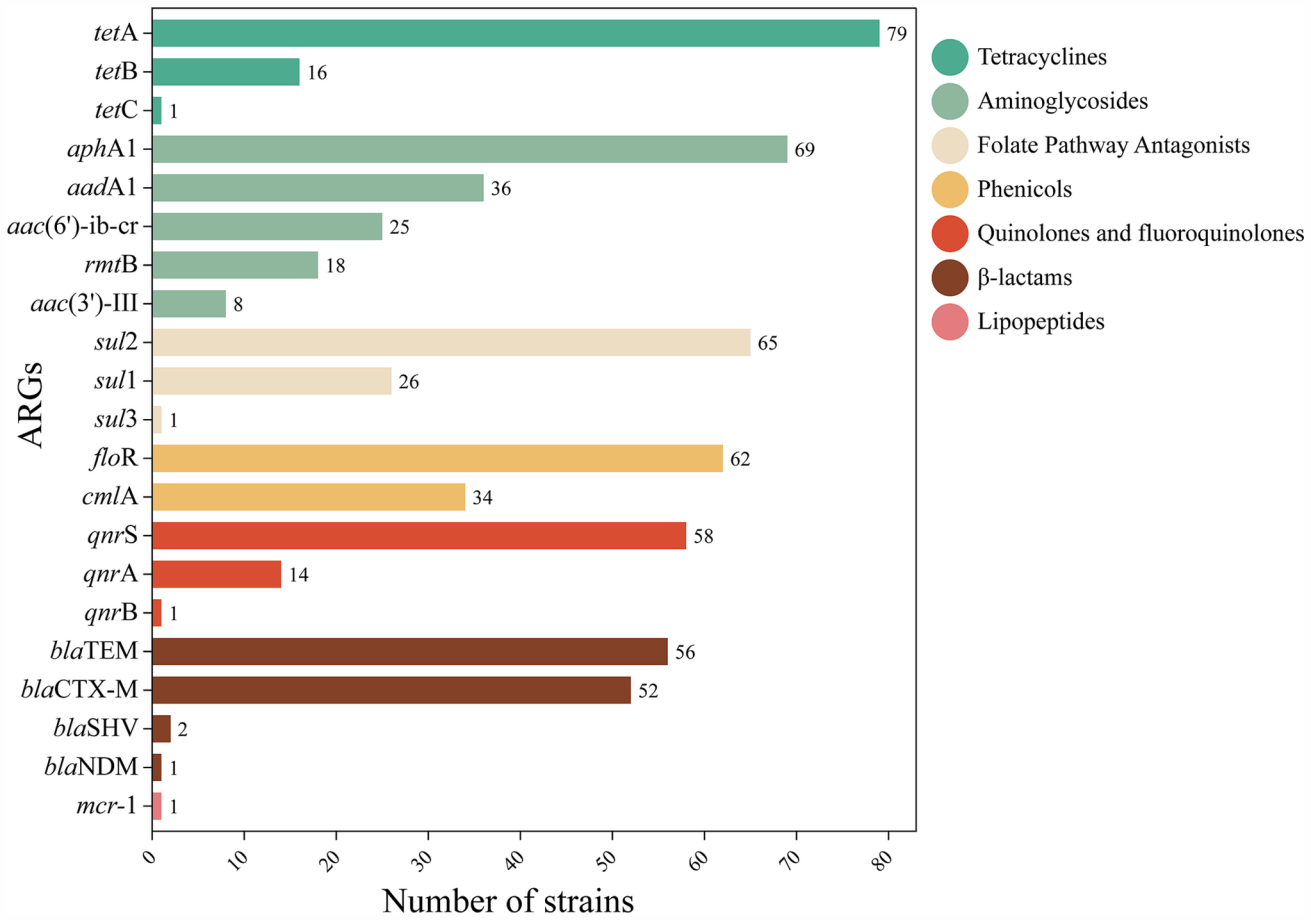
Distribution of number of ARGs. The distribution and quantity of different ARGs in FOS resistant strains were described. Color codes represent different classes of antimicrobial. Each bar represents a specific gene, and its length represents the number of genes detected in strains.

Highly diverse ARGs were detected in the genomes of the isolated strains, suggesting that waterfowl have the potential to serve as a reservoir of ARGs. Of the 26 ARGs, 21 were detected (Figure 2), with the major genotypes including: tetracyclines: *tetA* (79.0%); phenicols: *floR* (62.0%); quinolones: *qnrS* (58.0%); sulfonamides: *sul2* (65.0%); aminoglycosides: *aphA1* (69.0%); and *β*-lactams: *bla*_TEM_ (56.0%). Other ARGs were distributed in the following proportions: tetracyclines (*tet*B, 16.0%; *tet*C, 1.0%), phenicols (*cmlA*, 34.0%), quinolones (*qnrA*, 14.0%; *qnrB*, 1.0%), sulfonamides (*sul1*, 26.0%; *sul3*, 1.0%), aminoglycosides (*aadA1*, 36.0%; *aac(6′)-ib-cr*, 25.0%; *rmtB*, 18.0%; *aac(3′)-III*, 8.0%), β-lactams (*bla*_CTX-M_, 52.0%; *bla*_SHV_, 2.0%; *bla*_NDM_, 1.0%), peptides (*mcr*-*1*. 1.0%).

The *χ*^2^-test was utilized to investigate the association between ARGs and AMR. The findings indicated that the *bla*_CTX-M_ was significantly correlated with β-lactams resistance (*p* = 0.014), the *tetA* had a significant correlation with tetracyclines resistance (*p* = 0.001), the *floR* exhibited a significant correlation with phenicols resistance (*p* = 0.028), and the *sul2* displayed a significant correlation with sulphonamides resistance. Moreover, Supplementary Table S3 summarizes the prevalence of ARGs and their associations in waterfowl-derived FOS-resistant *E. coli*. Statistical analysis revealed multiple noteworthy correlations (*p* < 0.05) among these ARGs, with positive correlations being more prevalent, totaling 16 pairs of positive correlations and 11 pairs of negative correlations, resulting in a total of 27 pairs. of particular note is the strongest positive correlation observed between *bla*_CTX-M_ and *fos*A3 (OR, 15.162; 95% CI, 1.875–122.635).

### Molecular typing analysis of FOS-resistant *Escherichia coli*

**Figure 3:**
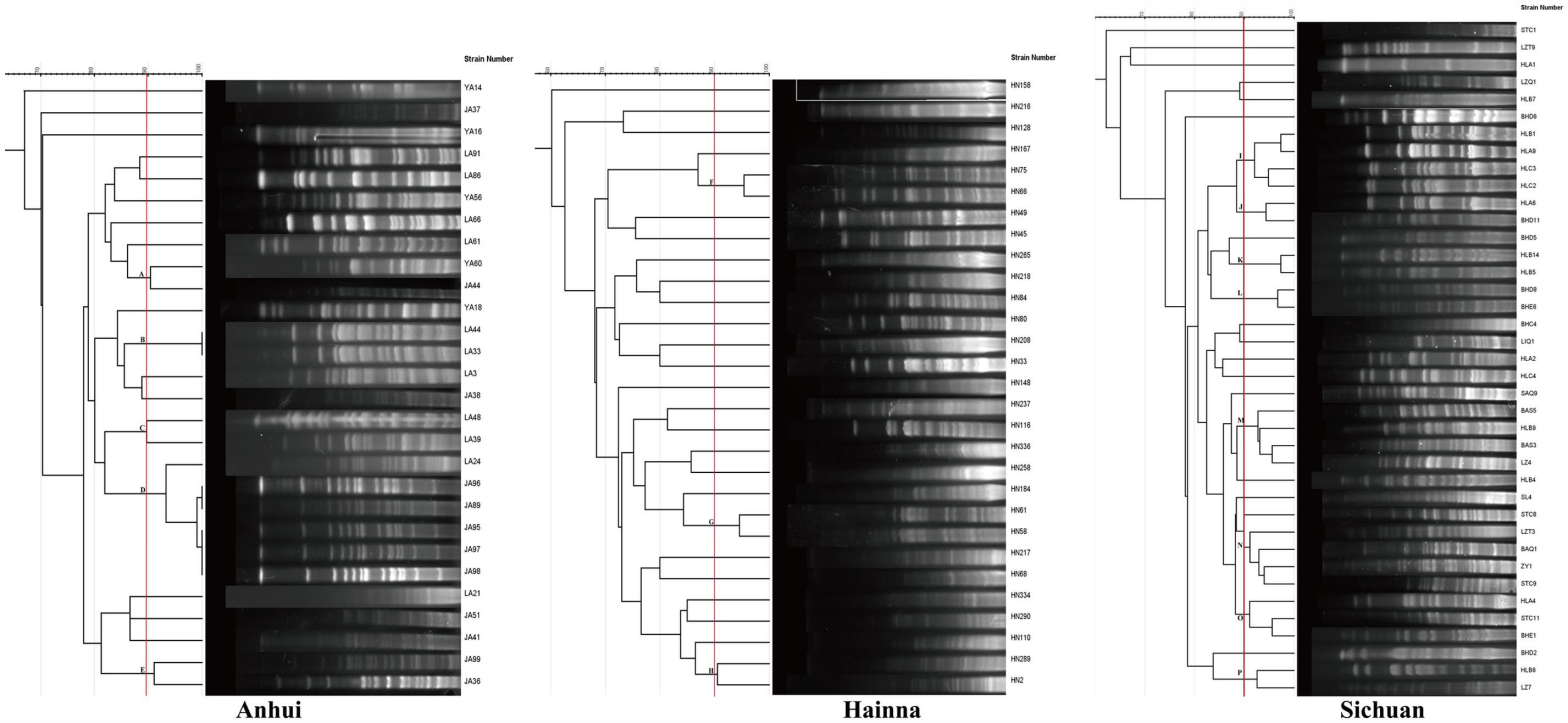
Strain typing based on PFGE. The figure shows the molecular typing of strains from Anhui, Hainan and Sichuan by PFGE. The strains in each region were clustered according to similarity, and their genetic relationships were displayed through a tree view. The bright colored bands in the figure represent DNA fragments, and the pattern of bands can reflect the genetic similarity between strains.

**Figure 4:**
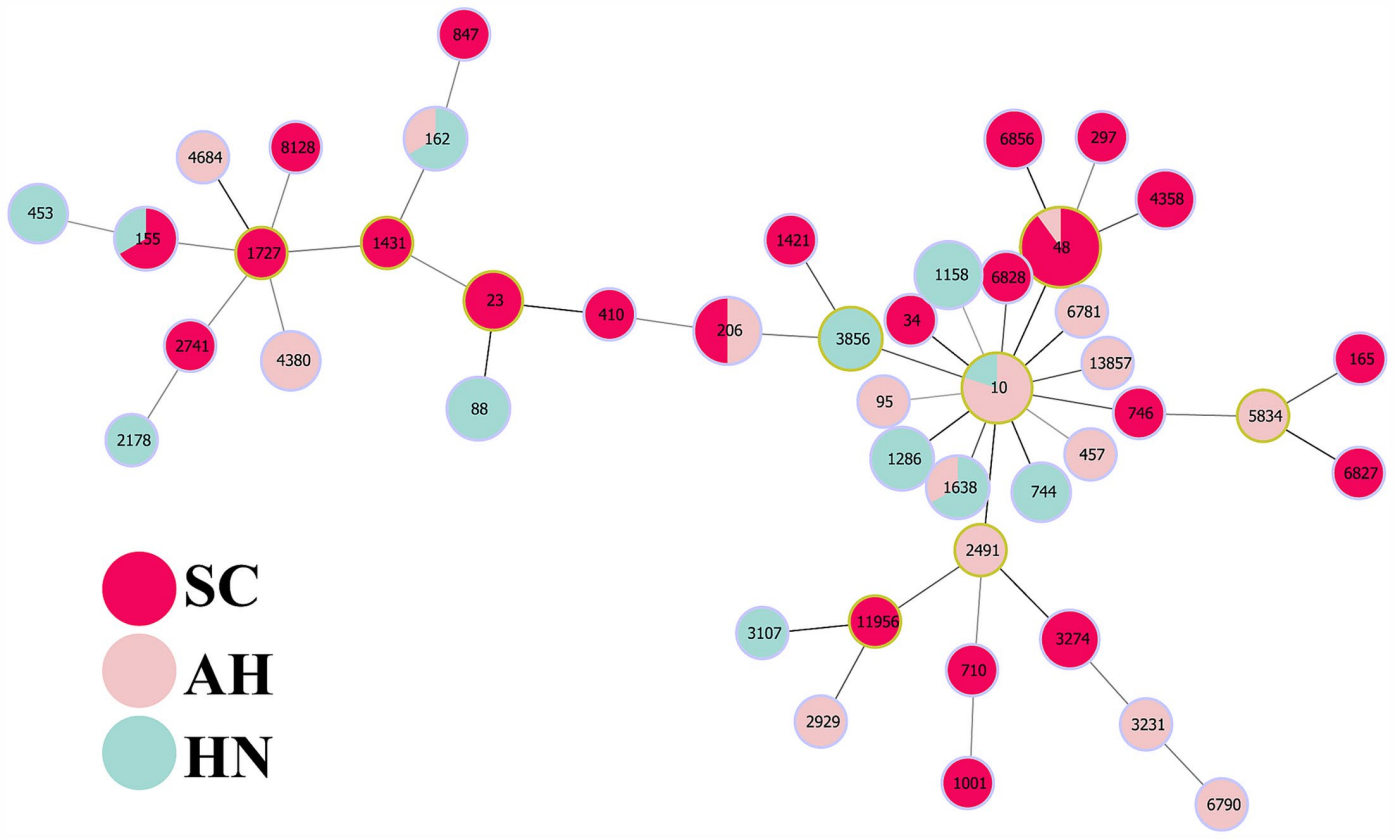
MLST minimum spanning tree of FOS resistant strains. This figure shows the minimum spanning tree of MLST of MDR FOS resistant strains. Nodes represent different sequence types, the size of nodes represents the number of strains of the sequence type, and the line between nodes represents the genetic distance between sequence types. Nodes with different colors represent different regions: red (SC), pink (AH), lightcyan (HN).

In order to investigate whether the spread of FOS resistance is closely related to clonal transmission, 100 FOS-resistant strains were subjected to PFGE typing in this study. The results demonstrated that 96 strains were successfully typed, including 39 strains from Sichuan, 28 strains from Anhui, and 29 strains from Hainan (Figure 3). 4 strains were unable to be typed. The evolutionary tree of PFGE profiles, constructed using Gel Jv 2.0 software, was employed to identify strains with greater than 90% similarity of PFGE profiles, which were then designated as belonging to the same clonal group. In the Anhui region, 5 clonal groups (A–E) were identified among 28 strains, all of which exhibited greater than 90% similarity. Among these, group D comprised 6 clonal strains. 3 clonal groups (F, G, and H) were identified among the 29 strains in the Hainan region, with similarities exceeding 90.0%. The remaining strains exhibited a PFGE spectral similarity that was primarily distributed between 70.0 and 85.0%. In Sichuan, 8 clonal groups (I–P) were identified among 39 strains, with the PFGE spectral similarity of the remaining strains concentrated in the range of 80.0–90.0%. The PFGE analysis revealed that the transmission of FOS resistance was clonally transmitted in the 3 regions, although this was not the main mode of transmission. The MLST results showed that the ST types of 83 strains were identified, involving a total of 45 ST types. The most prevalent types were ST48 (*n* = 10), ST10 (*n* = 5), ST206 (*n* = 4), and ST115 (*n* = 4). The minimum spanning tree constructed using Phyloviz 2.0 software demonstrated that these strains primarily formed 2 major clusters, centered on ST10 and ST1727, respectively. The genetic distances between the strains in Sichuan were found to be more closely related than those in Anhui and Hainan (Figure 4).

The 100 strains were classified into 7 phylogenetic clusters, indicating that FOS-resistant bacteria have diverse phylogenetic backgrounds. In particular, 36 strains were assigned to cluster A, 18 strains to cluster B1, 7 strains to cluster B2, 17 strains to cluster C, 9 strains to cluster D, and 10 strains to cluster E. Additionally, 1 strain was classified as belonging to cluster F, 1 strain was unassigned in Anhui, and 1 strain was assigned to branch I/II in Sichuan. These findings provide an important basis for understanding the mechanisms of FOS resistance transmission and for the development of prevention and control strategies.

### Characterisation of horizontal transmission of the fosfomycin resistance

**Figure 5:**
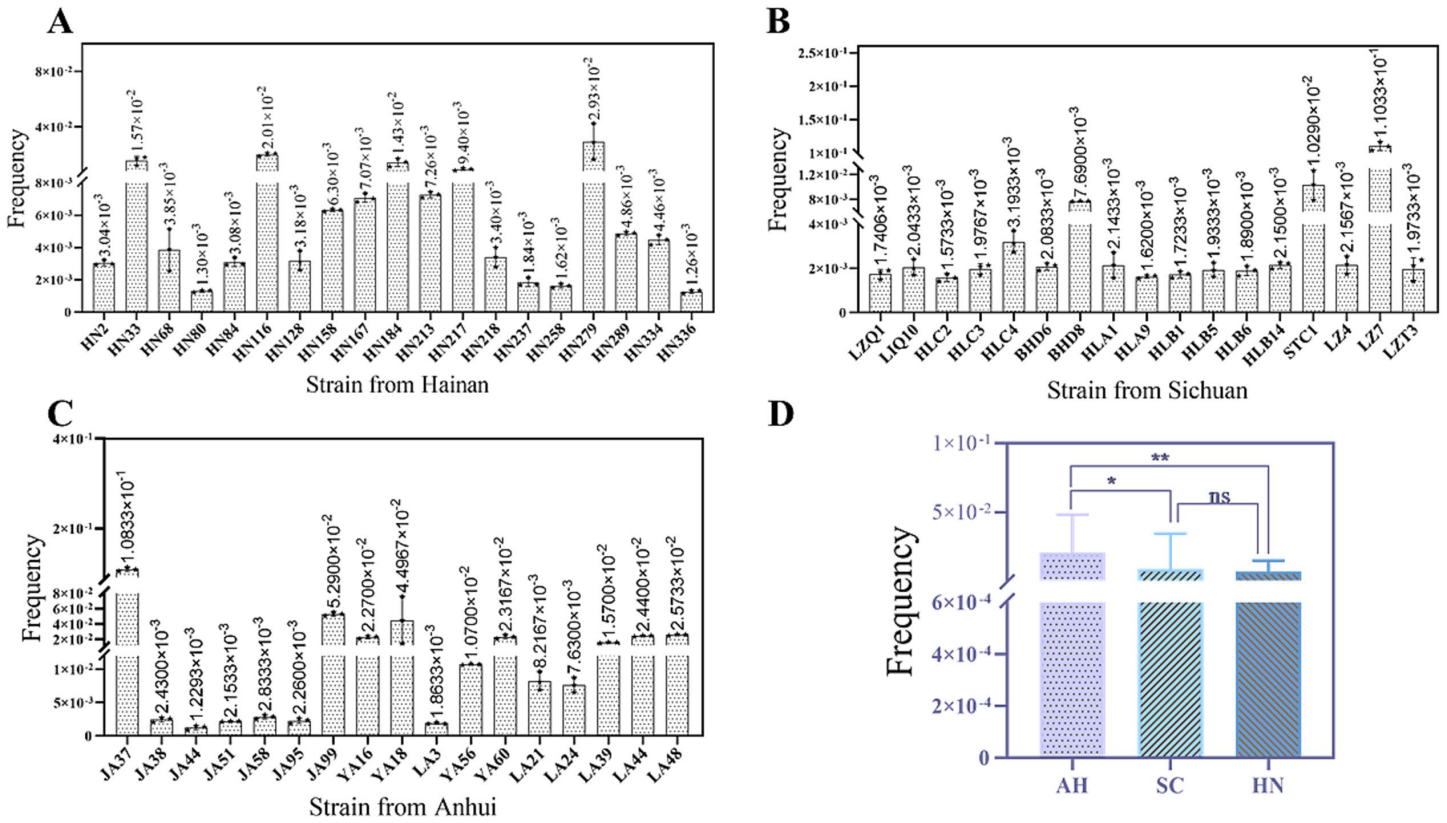
Conjugation transfer frequency of transconjugants in different regions. This figure shows the conjugation transfer frequency of 53 transconjugants. **(A)** The transfer frequency of transconjugants in Hainan; **(B)** The transfer frequency of transconjugants in Sichuan; **(C)** The transfer frequency of transconjugants in Anhui; **(D)** Comparison of transfer frequency in 3 regions. In panels **(A-C)**, the histogram in the figure shows the transfer frequency of each transconjugants, and the error line represents the standard error. In panel **(D)**, asterisk indicates statistical significance: *indicates *P* < 0.05, **indicates *P* < 0.01, and NS indicates no significant difference.

The plasmid conjugation transfer assay yielded 53 *fos*A-like gene positive transconjugants, representing a success rate of 53.0%. The conjugation transfer frequencies of the 53 transconjugants under investigation ranged from 1.3 × 10^−3^ to 1.1 × 10^−1^ (Figure 5). Among the various locations, the conjugation transfer frequencies of the transconjugants in Anhui were found to be significantly higher than those in Sichuan and Hainan. MICs of FOS for all transconjugants were ≥ 512 mg/L, and transconjugants exhibited MDR. In addition, other types of ARGs were found to be co-transferred in the transconjugants, including *aadA*1, *cmlA*, *floR*, *bla*_CTX-M_, *bla*_TEM_, *qnrS*, *sul1*, *sul2*, and *tet*A (Table 2).

**Table 2:** Comparison of ARGs and AMR of transconjugants.

Transconjugants	ARGs	AMR
T-LZQ1	*fos*A3/*bla*_CTX-M_/*qnr*S/*tet*A	OFX/NFX/CTX/AMP/FLM/TET/FOS
T-HLC2	*fos*A3/*bla*_CTX-M_/*flo*R/*qnr*S/*tet*A	CTX/AMP/FLM/SMZ/FOS
T-HLC3	*fos*A3/*qnr*S/*tet*A	OFX/NFX/CTX/AMP/FLM/TET/FOS
T-HLC4	*fos*A3/*bla*_CTX-M_/*bla*_TEM_	CTX/AMP/CAZ/FLM/TET/SMZ/FOS
T-BHD6	*fos*A3/*bla*_CTX-M_/*bla*_TEM_	CTX/AMP/FLM/TET/FOS
T-BHD8	*fos*A3/*aad*A1	CTX/AMP/FLM/SMZ/FOS
T-HLA1	*fos*A3/*bla*_CTX-M_/*flo*R/*qnr*S/*tet*A	CTX/AMP/FLM/TET/SMZ/FOS
T-HLA9	*fos*A3/*aad*A1/*bla*_CTX-M_/*flo*R/*qnr*S/*sul*1/*sul*2/*tet*A	OFX/NFX/CTX/AMP/FLM/TET/FOS
T-HLB1	*fos*A3/*bla*_TEM_/*sul*1/*sul*2/*tet*A	NFX/CTX/TET/SMZ/FOS
T-HLB5	*fos*A3/*flo*R/*qnr*S/*sul*1/*tet*A	CTX/AMP/FLM/TET/SMZ/FOS
T-HLB6	*fos*A3/*aad*A1/*bla*_TEM_/*flo*R/*qnr*S/*sul*1/*tet*A	OFX/CTX/AMP/FLM/TET/SMZ/FOS
T-HLB14	*fos*A3/*aad*A1/*flo*R/*qnr*S/*tet*A	CTX/AMP/FLM/TET/SMZ/FOS
T-STC1	*fos*A3/*cml*A/*bla*_CTX-M_/*bla*_TEM_/*flo*R/*sul*2/*tet*A	AMK/GEN/CTX/AMP/CAZ/FLM/TET/FOS
T-LZ4	*fos*A3/*bla*_CTX-M_/*flo*R/*qnr*S/*tet*A	CTX/AMP/FLM/TET/SMZ/FOS
T-LZ7	*fos*A3/*bla*_CTX-M_/*bla*_TEM_/*tet*A	CTX/AMP/CAZ/FOS
T-LZT3	*fos*A3/*bla*_TEM_/*flo*R/*qnr*S/*tet*A	OFX/NFX/CTX/AMP/FLM/TET/FOS
T-JA37	*fos*A3/*aad*A1/*bla*_CTX-M_	CTX/AMP/CAZ/FLM/FOS
T-JA38	*fos*A3/*bla*_CTX-M_/*qnr*S	CTX/AMP/CAZ/FOS
T-JA44	*fos*A3/*bla*_TEM_/*flo*R/*tet*A	AMP/FLM/TET/FOS
T-JA51	*fos*A3/*bla*_CTX-M_/*flo*R	CTX/AMP/TET/FOS
T-JA58	*fos*A3/*flo*R/*sul*2	CTX/AMP/CAZ/FLM/TET/IMP/SMZ/FOS
T-JA95	*fos*A3/*bla*_TEM_/*flo*R/*sul*2	CTX/AMP/CAZ/FLM/TET/IMP/SMZ/FOS
T-JA99	*fos*A3/*bla*_TEM_/*flo*R/*tet*A	AMP/FLM/TET/SMZ/FOS
T-YA16	*fos*A3/*aad*A1/*bla*_CTX-M_/*flo*R	CTX/AMP/SMZ/FOS
T-YA18	*fos*A3/*aad*A1/*qnr*S/*sul*2/*tet*A	CTX/AMP/FLM/TET/SMZ/FOS
T-YA56	*fos*A3/*flo*R/sul1/*tet*A	CTX/AMP/FLM/TET/TGC/FOS
T-YA60	*fos*A3/cmlA/*bla*_TEM_/*tet*A	AMK/GEN/CTX/AMP/SMZ/FOS
T-LA21	*fos*A3/*cml*A/*bla*_CTX-M_/*flo*R/*qnr*S/*sul*2/*tet*A	CTX/AMP/FLM/TET/FOS
T-LA24	*fos*A3/*qnr*S/*tet*A	NFX/CTX/AMP/FLM/TET/PB/FOS
T-LA39	*fos*A3/*cml*A/*bla*_TEM_/*tet*A	CTX/AMP/FLM/TET/SMZ/FOS
T-LA44	*fos*A3/aadA1/*cml*A/*bla*_TEM_/*flo*R/*tet*A	CTX/AMP/FLM/TET/SMZ/FOS
T-LA48	*fos*A3/*bla*_CTX-M_/*flo*R/*tet*A	CTX/AMP/FLM/TET/FOS
T-HN2	*fos*A6+*fos*A10/cmlA/CTX-M	CTX/AMP/TGC/SMZ/FOS
T-HN33	*fos*A3/*bla*_CTX-M_/*sul*1/*sul*2/*tet*A	GEN/AMP/TET/SMZ/FOS
T-HN68	*fos*A3/*bla*_CTX-M_/*tet*A	CTX/AMP/SMZ/FOS
T-HN80	*fos*A3/*bla*_CTX-M_	CTX/AMP/CAZ/FLM/FOS
T-HN84	*fos*A3/*bla*_CTX-M_/*bla*_TEM_/*flo*R/*sul*2/*tet*A	CTX/AMP/TET/SMZ/FOS
T-HN116	*fos*A3/*bla*_CTX-M_/*qnr*S/*sul*1	CTX/AMP/CAZ/TET/SMZ/FOS
T-HN128	*fos*A3/*bla*_CTX-M_/*flo*R/*sul*2/*tet*A	CTX/AMP/FLM/TET/FOS
T-HN158	*fos*A3/*bla*_CTX-M_/*sul*2/*tet*A	CTX/AMP/FLM/TET/PB/SMZ/FOS
T-HN167	*fos*A3/*bla*_CTX-M_/*flo*R/*sul*2/*tet*A	CTX/AMP/FLM/TET/SMZ/FOS
T-HN184	*fos*A3/*bla*_CTX-M_/*qnr*S	CTX/AMP/CAZ/TET/SMZ/FOS
T-HN213	*fos*A3/*flo*R/*qnr*S	CTX/AMP/FLM/TET/PB/SMZ/FOS
T-HN217	*fos*A3/*bla*_CTX-M_/*flo*R/*sul*2/*tet*A	CTX/AMP/CAZ/FLM/TET/SMZ/FOS
T-HN218	*fos*A3+*fos*A10/*bla*_TEM_/*flo*R/*sul*2/*tet*A	GEN/CTX/AMP/FLM/TET/FOS
T-HN237	*fos*A3/*cml*A/*bla*_TEM_	AMP/CAZ/FLM/SMZ/FOS
T-HN258	*fos*A3/*cml*A/*bla*_TEM_/*qnr*S	GEN/CTX/AMP/FOS
T-HN279	*fos*A3/*bla*_CTX-M_	CTX/AMP/CAZ/SMZ/FOS
T-HN289	*fos*A3/*bla*_TEM_	AMK/GEN/CTX/AMP/CAZ/SMZ/FOS
T-HN334	*fos*A3/*cml*A/*bla*_TEM_	AMK/GEN/CTX/AMP/CAZ/SMZ/FOS
T-HN336	*fos*A3/*aad*A1/*bla*_CTX-M_	AMK/GEN/CTX/AMP/FOS
T-LA33	*fos*A3/*aad*A1/*bla*_TEM_/*flo*R/*tet*A	AMP/IMP/FLM/TET/FOS
T-LIQ10	*fos*A3/*qnr*S/*tet*A	AMP/FLM/TET/FOS

The plasmid carriage of the 53 transconjugants and their corresponding donor bacteria was investigated. The findings revealed that a total of 16 distinct plasmid replicon types were carried by the 53 donor bacteria, with the majority plasmid replicon types falling into the FIB and frepB (Table 3).

**Table 3:** Plasmid replicons of the FOS-resistant ***E. coli*** and their transconjugants.

Strains	Plasmid replicon types	Transconjugants	Plasmid replicon types	Strains	Plasmid replicon types	Transconjugants	Plasmid replicon types
LZQ1	Y, K	T-LZQ1	k	YA56	FIB	T-YA56	FIB
LIQ10	K	T-LIQ10	k	YA60	FIB	T-YA60	FIB
HLC2	FIB, K	T-HLC2	k	LA21	FIA, FIB, W	T-LA21	FIA, W
HLC3	I1, N, Y, FIC	T-HLC3	I1, FIC	LA24	FIA, FIB, W, N	T-LA24	FIA, FIB, W
HLC4	I1, FIC, N	T-HLC4	I1, FIC	LA39	HI2, FIB, I1	T-LA39	HI2
BHD6	FIB, FII, X4	T-BHD6	FII	LA44	FIB, A/C, frepB	T-LA44	frepB
BHD8	I1, N, frepB	T-BHD8	I1, N	LA48	HI2, FIB	T-LA48	HI2
HLA1	HI2, N, A/C, B/O	T-HLA1	HI2, N	HN2	FIB, T	T-HN2	FIB, T
HLA9	I1, N, frepB	T-HLA9	I1, N	HN33	FIA, W, P, frepB	T-HN33	FIA, P
HLB1	A/C, FIB	T-HLB1	FIB	HN68	FIB, FII	T-HN68	FIB, FII
HLB5	A/C, FIB	T-HLB5	FIB	HN80	FIB, N, Y	T-HN80	FIB
HLB6	I1, N, Y, FIC	T-HLB6	FIC	HN84	FIB, T	T-HN84	FIB
HLB14	A/C	T-HLB14	A/C	HN116	FIA, FIB, W	T-HN116	FIB, W
STC1	I1, N, Y, FIB	T-STC1	I1, N	HN128	FIB, A/C	T-HN128	FIB
LZ4	FIB, frepB	T-LZ4	FIB	HN158	FIB, Y	T-HN158	Y
LZ7	FIB, frepB	T-LZ7	FIB	HN167	FIB, Y, A/C	T-HN167	FIB, Y
LZT3	HI1, FIB, Y	T-LZT3	FIB	HN184	FIB, frepB	T-HN184	frepB
JA37	FIB, frepB	T-JA37	FIB	HN213	HI1, FIB, A/C	T-HN213	HI1
JA38	FIB, Y, frepB	T-JA38	FIB, frepB	HN217	FIB, Y, A/C	T-HN217	FIB, A/C
JA44	HI1, FIB, B/O	T-JA44	FIB	HN218	FIA, FIB, W, A/C	T-HN218	FIB, A/C
JA51	N, frepB	T-JA51	frepB	HN237	HI1, FIB, A/C	T-HN237	HI1
JA58	frepB	T-JA58	frepB	HN258	HI1, FIB, B/O, I1	T-HN258	I1
JA95	FIA, W, frepB	T-JA95	FIA, W, frepB	HN279	Y, P, I1	T-HN279	I1
JA99	FIA, W, frepB	T-JA98	FIA, W, frepB	HN289	FIA, FIB, Y, T	T-HN289	FIA
YA16	frepB	T-YA16	frepB	HN334	FIA, FIB, W, Y, T, I1	T-HN334	FIB, I1
YA18	FIB, W, frepB	T-YA18	FIA, W, frepB	HN336	FIA, FIB, W, Y, T	T-HN336	FIB
LA3	HI2, FIB, X1	T-LA3	FIB, X1				

### Analysis of *fos*A3-positive plasmids

**Figure 6:**
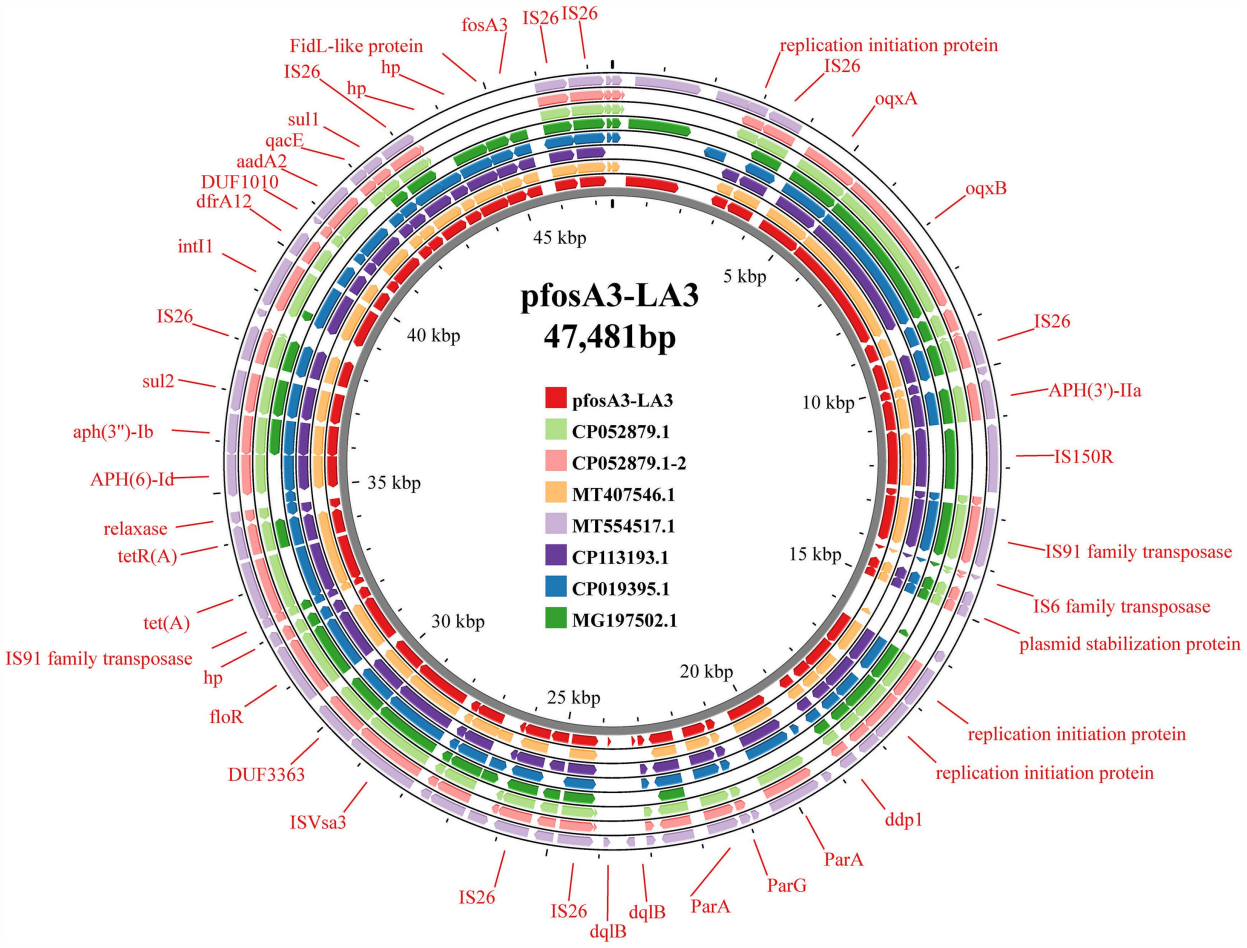
Comparison diagram of pfosA3-LA3 plasmid and other similar plasmids. The figure shows the comparison between pfosA3-LA3 plasmid and other similar plasmids. The ring chart in the figure shows the genome structure of each plasmid, and different colors represent different plasmids. The illustration highlights key genes and functional areas, including replication initiation proteins, transposases, and ARGs.

**Figure 7:**
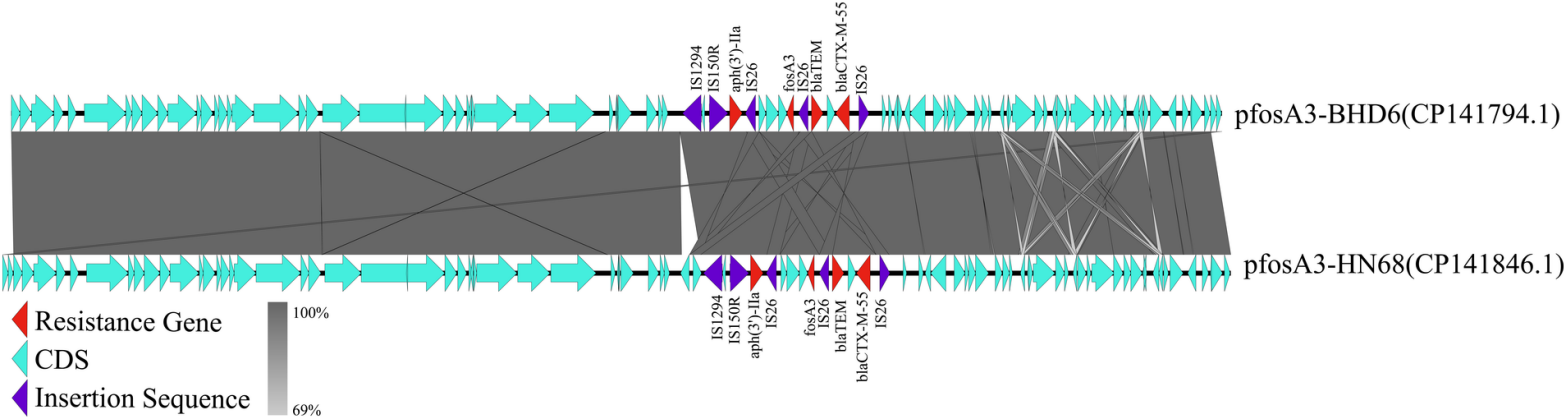
Co-linearity analysis of plasmid pfosA3-BHD6 with plasmid pfosA3-HN68. In the figure, different colors and shapes of markers are used to represent different genes and sequence characteristics: the red triangle represents the ARGs, the light blue arrow represents the coding DNA sequence (CDS), and the purple diamond represents the insertion sequence. The gray shaded area indicates the degree of collinearity between the 2 plasmids, and the line connection indicates the homology between genes. This figure reveals the similarities and differences between the 2 plasmids in genome structure.

In strain LA3, the *fos*A3 was localized in the IncX1-type plasmid p*fos*A3-LA3 (47,481 bp, CP141843.1), which lacks the type IV secretion system but contains genes related to stabilizing, replicating and relaxase (Figure 6). This plasmid is very similar to the *Enterobacteriaceae* IncX1 plasmid, but only partially carries the *fos*A3, the transfer of which may be related to the IS*26* insertion sequence. In HN68 and BHD6, the *fos*A3 was located within the IncFII (F16:A-:B) type plasmids, p*fos*A3-HN68 (77,578 bp, CP141846.1) and p*fos*A3-BHD6 (76,461 bp, CP141794.1), respectively, with a GC content of 52.6%. Both contained a MDR region (12.3 Kbp) with 4 ARGs and multiple insertion sequences, and shared a 40.4 Kbp plasmid backbone region containing replication, conjugation-transfer and relaxase-related genes. The 2 were highly similar (99.0% coverage, 100.0% similarity) but differed in ST type and serotype, suggesting that they were from different sources (Figure 7).

**Figure 8:**
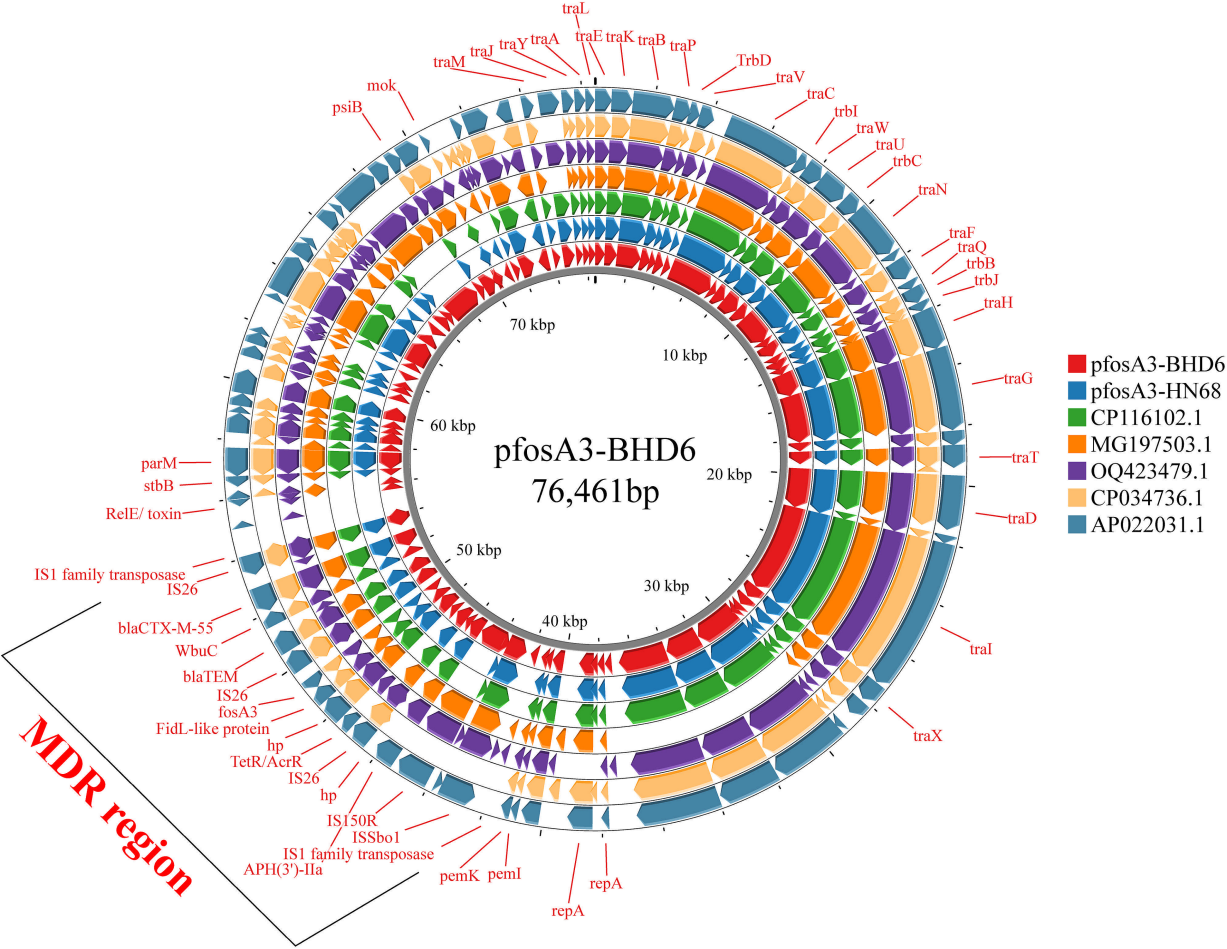
Comparison of plasmids pfosA3-BHD6 and pfosA3-HN68 with other similar plasmids. It illustrates the genomic similarities and differences among these plasmids. The different colors in the figure represent distinct plasmids, with key genes and the MDR region highlighted. The MDR region contains multiple ARGs.

Comparison with the NCBI database revealed that p*fos*A3-HN68 and p*fos*A3-BHD6 were highly similar (>85.0% coverage, >90.0% similarity) to several *E. coli* plasmids in which the *fos*A3 coexisted with elements such as IS*26* and *bla*_TEM_ on a genomic island, allowing further study of the distribution of the *fos*A3 in *E. coli* plasmids from different sources and the propagation of the *fos*A3 in different sources of *E. coli* plasmids (Figure 8).

### Analysis of the genetic environment of the *fos*A3 gene

**Figure 9:**
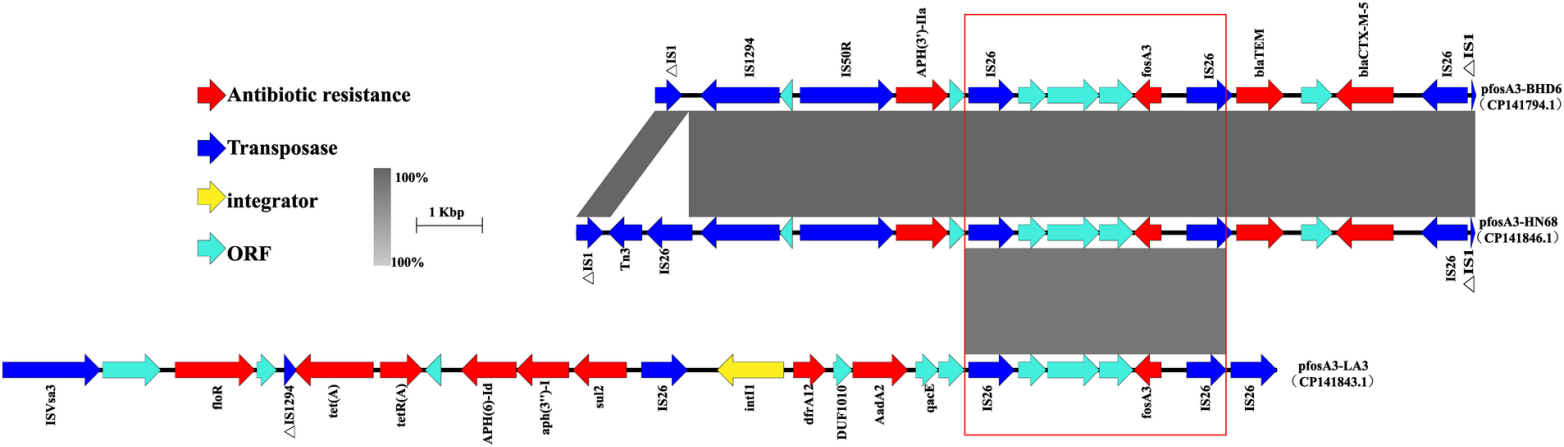
Genetic structure of the *fosA3* environment. In the figure, different colors and shapes of markers are used to represent different genes and sequence characteristics: the red arrow represents the ARG, the blue arrow represents the transposase gene, the yellow diamond represents the integron, and the light blue arrow represents the ORF. The gray shaded area indicates the high similarity area, and the part marked by the red box shows the conservative structure near the *fos*A3 in different plasmids.

The 3 *fos*A3-positive plasmids share a common *fos*A3 core genetic environment surrounding the *fos*A3. The gene is flanked by IS*26* both upstream and downstream. Notably, the IS*26* sequences upstream of *fos*A3 are identical at 385 bp in length, whereas the downstream IS*26* encompasses 3 open reading frames (ORFs) situated between its 3′ end and the IS*26* element. These ORFs encode transcriptional regulators belonging to the TetR/AcrR family, hypothetical proteins, and FidL-like proteins, respectively. Collectively, the 2 IS*26* elements may constitute a composite transposon that promotes the horizontalTransconjugantssfer of the *fos*A3 (Figure 9).

In plasmids p*fos*A3-BHD and p*fos*A3-HN68, there is a MDR region near the *fos*A3, accompanied by the ESBL genes *bla*_TEM_ and *bla*_CTX-M-55_ upstream and the aminoglycoside resistance gene *aph* (3′)-IIa downstream, surrounded by the IS*50R*, IS*Sbo1* and the truncated IS*1* family insertion element, forming a stable genetic structure. In plasmid p*fos*A3-HN68, transposons Tn*3* and IS*26* were inserted between ΔIS*1* and IS*1294*.

In plasmid p*fos*A3-LA3 there is a type I integron structure downstream of the *fos*A3, containing gene cassettes such as the folate pathway inhibitor resistance gene *dfrA12*, the aminoglycoside resistance gene *aadA2* and the structural domain protein DUF1010. The 3′ end of the integron consists of the quaternary ammonium efflux transporter protein *qacE* and the truncated folate pathway inhibitor *sul1*, surrounded by IS*26*. Downstream of the integrase gene *intI*1 is the multidrug resistance region, consisting of IS*26*, IS*Vs*A3 and a number of ARGs such as *floR*, *tet*(A), *aph* (6)*-Id*, *aph(3″)-Ib*, *sul2*, etc., which together form a complex drug resistance network.

## Discussion

### Characterization of resistance in FOS-resistant *Escherichia coli*

Despite the prohibition of FOS in veterinary medicine in China since 2005, a survey of 833 *E. coli* strains of waterfowl origin from Hainan, Sichuan and Anhui revealed that the resistance rate to FOS remained alarmingly high at 12.0%. Among the 100 resistant strains, *fos*A3 exhibited the highest positivity rate (88.0%). This finding aligns with previous studies, indicating that *fos*A3 is the most prevalent plasmid-mediated resistance gene for FOS (30). The *fos*A3 positive *E. coli* were detected across various sample types; Notably, a detection rate of 21.9% was observed in *E. coli* sourced from ducks in Shandong (31), while a positive rate of 27.4% was reported for *E. coli* isolated from poultry farms (32). The tested strains demonstrated significantly high levels of resistance to FOS (MIC≥512 mg/L) and varying degrees of resistance to an additional 14 antimicrobial agents. Notably, high levels of resistance were observed against AMP, FLM, CTX and TET indeed, 99.0% of these strains were classified as MDR bacteria. MDR means resistance to 3 or more unique antimicrobial drug classes (33, 34). This elevated level of AMR may be closely associated with environmental contamination stemming from residual tetracyclines, phenicols and sulfonamides antimicrobials (35).

It has been demonstrated that waterfowl farm waste contains a significant amount of ARGs, which pose potential threat to public health and the environment (12). The importance of ARGs, which confer bacterial resistance to antimicrobials, cannot be overstated. They represent a considerable challenge for antimicrobial therapy at present (36). In this study, 26 ARGs were subjected to comprehensive testing and analysis. The study revealed that the prevalence of *tet*A, *aphA1*, *sul2*, *folR*, *qnrS*, and *bla*_TEM_ in *E. coli* derived from waterfowl was significantly elevated. It was demonstrated that high levels of veterinary tetracyclines and sulfonamides discharged into the environment substantially contributed to the widespread presence of *tet* and *sul* ARGs (35). Further analyses revealed that although there were differences between bacterial resistance phenotypes and ARGs, some genes exhibited significant associations with specific resistance phenotypes. In particular, *bla*_CTX-M_ was found to be significantly associated with *β-lactams* resistance, *tet*A with tetracyclines resistance, *floR* with phenicols resistance, and *sul2* with sulfonamides resistance (*p* < 0.05). These studies have attributed this phenomenon to abnormal expression or low expression levels of ARGs (37). The present study demonstrated a significant positive correlation between the *fos*A3 and *tet*A, *qnrS*, and *bla*_CTX-M_, as well as *fos*A3 frequently coexisted with these ARGs (7, 38). However, the *fos*A3 exhibited a negative correlation (OR < 1) with *rmtB* (39). Given the extensive use of tetracyclines, β-lactams, aminoglycosides, and chloramphenicols in Chinese animal husbandry, it is plausible that *fos*A3 may coexist alongside multiple ARGs, such as *tet*A, *qnrS*, *bla*_CTX-M_, and *folR*, within the same strain. This may, in turn, facilitate the dissemination of *fos*A3 (40). Despite the explicit prohibition on FOS as a veterinary drug in China, the co-existence of plasmid-borne *fos*A3 alongside other ARGs may have contributed to the observed increase of FOS resistance in bacteria of animal origin and the widespread dispersal of the *fos*A3 under co-selection pressure (41).

### Molecular typing analysis of *fos*A-like-positive *Escherichia coli*

This study subjected 100 FOS-resistant strains to PFGE typing. The resulting data demonstrated that Anhui, Hainan, and Sichuan were distinctly different. While the majority of strains exhibited unique PFGE profiles, these findings nonetheless underscore a significant clonal spread characteristic of FOS-resistant strains. Notably, this phenomenon of clonal spread was particularly conspicuous in Sichuan. Several studies have highlighted the potential role of migratory birds and poultry exposure to wildlife in facilitating cross-regional transmission of AMR (42). One study has shown that even in the absence of ARGs contamination at hatcheries, the introduction of ARGs into the flock can occur rapidly through vectors such as dogs, flies, wild birds, and others (43). Our previous research has shown that waterfowl farming in open environments can also lead to the development of antibiotic resistance among carrying bacteria (23). Thus, inadequate environmental control and biosecurity measures on farms may facilitate the dissemination of FOS-resistant bacteria and *fos*A3 among waterfowl.

In epidemiological investigations, MLST and PFGE are commonly employed methods for the molecular typing of *E. coli* (44). MLST analysis effectively classified 83 *E. coli* strains into 45 known sequence types (STs), with ST48 (*n* = 10) and ST10 (*n* = 5) being the most prevalent types. Notably, all 10 ST48 strains were found to be positive for the *fos*A3 and originated from Sichuan Province. PFGE analysis revealed that some strains belonged to the same clonal group, suggesting a clonal spread within Sichuan; however, this was not indicative of a broader trend. The geographical distribution of ST types s across three regions was diverse, with strains sharing the same ST type potentially belonging different PFGE clonal groups. Reports on *fos*A3-positive *E. coli* strains associated with ST48 and ST10 are rare in existing literature. A previous study indicated that the most prevalent type of *fos*A3-positive *E. coli* sourced from waterfowl in Shandong was ST69 (31). Furthermore, ST156 and ST115 were the most prevalent types among *fos*A3-carrying *E. coli* of animal origin (10). Typically, *E. coli* ST48 isolates are associated with the spread of *bla*_NDM_ in poultry farms and retail meat (45). In contrast, *E. coli* ST10 is a low virulence commensal that is widely present in the animal and human intestinal tract (46). Furthermore, *E. coli* ST10 is regarded as a significant reservoir for the *mcr-1* (47). Among three identified *fos*A7-positive strains, three distinct STs were observed, such as ST847, ST1727, and ST2741; these have not been previously reported in the reference. Notably, although ST847 was previously reported in a *bla*_CTX-M_-positive plant-derived *E. coli* strain, it does not carry *fos*A-like genes (48). Moreover, out of 100 analyzed FOS-resistant strains categorized into seven phylogenetic groups, with groups A and B1 representing the majority, while groups B2 and D were the least numerous. The findings of the current study align with those of previous research conducted in Pakistan, which similarly identified groups B1 and A as the most prevalent strains of *E. coli* in both human and animal populations (49). Notably, the identification of an ST410 type D group *fos*A3-positive strain, as it is associated with ExPEC pathogens and represents a high-risk clonal group (50, 51).

### Analysis of horizontal transmission characteristics of *fos*A3 plasmid

Plasmids serve as a molecular cornerstone in the dissemination of AMR in the era of “One Health” (52). The first report of plasmid-mediated horizontal transfer of the *fos*A3 was published by Japanese researchers in 2010 (53). Subsequent studies have confirmed that the *fos*A3 is typically located on plasmids and spreads among various bacteria through horizontal transfer (7, 13). Despite the fact that FOS is not approved for use in veterinary clinics, the prevalence of the *fos*A3 remains significant across Asia (7). In this study, we found that the transconjugants from *fos*A3-positive strains transferred genes such as *bla*_CTX-M_, *floR*, and *aadA1* simultaneously during the plasmid conjugation transfer. This co-transfer enabled these strains to acquire resistance to cephalosporins, chloramphenicols, sulfonamides, and aminoglycosides, which are commonly used in veterinary clinics. CTX-Ms is at the pinnacle of the pyramid of ESBLs, which are distributed in different ecosystems and exhibit resistance to a broad spectrum of antibiotics (54). Due to the slow development of antibacterial agents, researchers are compelled to revisit the utilization of established antibiotics like FOS to combat MDR bacteria, particularly those within the *Enterobacteriaceae* family known for their ability to produce ESBLs and carbapenemases (55). However, prior research indicates that the *fos*A3 is generally co-located on plasmids with *bla*_CTX-M_, and they tend to co-transfer and disseminate together (56). *Fos*A3-positive *E. coli* were identified in pets that had not been treated with FOS. In these instances, it was found that the *fos*A3, flanked by IS*26* resided on an F33:A-:B-type plasmid also carrying *rmtB*. This suggests that transmission of the *fos*A3 is predominantly driven by horizontal transfer involving F33:A-:B- plasmid closely associated with *rmtB*, rather than through clonal transmission (57). Furthermore, co-transfer of the *floR* with the *fos*A3 was observed in chicken-derived *fos*A3-positive *E. coli* (40). The concerning co-occurrence of the *fos*A with key ARGs on plasmids presents an extra hurdle in treating *Enterobacteriaceae* infections (55). In this study, transconjugants of *fos*A and *fos*A7 positive strains could not be successfully obtained. The findings indicate that the *fos*A is frequently located on the chromosomes of various Gram-negative bacteria, including *Klebsiella pneumoniae*, *Streptococcus mucinosus*, and *Pseudomonas aeruginosa* (58). In contrast, the *fos*A7 is predominantly located on the chromosome of *Salmonella* (10). Furthermore, it was demonstrated that the *fos*A7 on the chromosome of *Salmonella* conferred a low level of resistance to FOS. However, the transfer of this gene into a plasmid resulted in a significant increase in resistance and fitness (59).

One of the key factors contributing to the spread of AMR is the high frequency of conjugation transfer. In this study, we observed that the conjugation transfer frequency of 53 transconjugants ranged from 1.3 × 10^−3^ to 1.1 × 10^−1^. This suggests that these plasmids possess a potential for dissemination in natural environments, particularly when their frequency approaches or exceeds values between 10^−1^ and 10^−2^ (60). This study identified multiple plasmid replicon types in the spliceosome, including FIB, FIA, frpB, HI1, HI2, and others. These findings indicate that the *fos*A3 can adapt to diverse plasmid environments encompassing both single-replicon and multi-replicon plasmids (9). Previous studies have demonstrated the prevalence of the *fos*A3 in a diverse range of plasmids, including the IncC-IncN-type plasmids (lacking horizontal transfer genes) of multidrug-resistant *Salmonella* (61). Additionally, the IncN-type and IncHI2-type plasmids of vegetable-derived *E. coli* were identified (48), as well as mucin-resistant *E. coli* harboring IncFII/FIA/FIB-type plasmids (62). Finally, coexisting with *bla*_CTX-M_ in chicken-derived *E. coli* were identified either IncHI2-type or IncI1-type plasmids (40). Furthermore, it was noted the *fos*A3 was disseminated in retail vegetable-derived ESBL-producing *E. coli* through various plasmid vectors, including IncHI2/ST3, IncN1-F33:A-:B-, F33:A-:B-, IncFIIK, and F24:A-:B1 (63). In conclusion, the rapid spread of FOS resistance in pathogenic bacteria of originating from animals is closely associated with the transfer of multiple plasmid-mediated resistance.

### Analysis of the characteristics of *fos*A3-positive plasmids

Sequencing results revealed that the *bla*_NDM-5_, *mcr*-*1*, and *bla*_CTX-M_ are all located on plasmids. However, in strains BHD6 and LA3, the *mcr-1* and *bla*_NDM-5_ do not coexist with *fos*A3 on the same plasmid. In contrast, in strains BHD6 and HN68, the *fos*A3 is located on the same plasmid as *bla*_CTX-M_. Notably, these two plasmids show high similarity and also carry other ARGs. The emergence of plasmid-mediated *mcr-1* and *bla*_NDM_ poses a serious threat to antibiotic treatment efficacy, presenting unprecedented challenges to public health and animal husbandry (64, 65). Although FOS retains some bactericidal activity against CRE, ESBL-EC, and colistin-resistant bacteria, its effectiveness can be severely compromised when coexisting with ARGs such as *bla*_NDM-5_, *mcr*-1, and *bla*_CTX-M_. This may lead to treatment failure. The presence of multiple resistance plasmids in a single strain frequently leads to resistance against nearly all available antibiotics while facilitating the dissemination of ARGs. Some studies have reported instances where *fos*A3, *mcr*-*1*, and *bla*_NDM_ coexist on a single plasmid, this phenomenon contributes to broad-spectrum resistance to multiple antibiotics in related strains (66–68).

In the 3 sequenced strains, the *fos*A3 and *bla*_CTX-M_ are located on typical IncFII plasmids, except for strain LA3. Previous studies have indicated that IncFII plasmids represent the primary type of plasmid harboring the *fos*A3 (9). Multiple studies have confirmed a strong association between the co-dissemination of *fos*A3 and *bla*_CTX-M_ on IncFII plasmids (69, 70), which is consistent with findings in the p*fos*A3-BHD6 and p*fos*A3-HN68 plasmids. Furthermore, sequence analysis results show that both p*fos*A3-BHD6 and p*fos*A3-HN68 plasmids contain multiple essential modules required for conjugative transfer, such as conjugative transfer protein TrbD, Type IV secretion system genes (including *tra* series and *tab* series), plasmid replication origin *ori*T, and relaxase enzyme. Plasmid conjugation experiments further confirm the horizontal transmission capability of these two plasmids. Although there are currently no specific research reports on *fos*A3-positive IncX1-type plasmids, several heterozygous plasmids carrying the *fos*A3 classified as IncX1 type have been identified in the NCBI database. For example, the heterozygous plasmid pXH992_2 (accession number CP019395.1) carrying the IncR-IncX1 type from urine samples of patients in Zhejiang, the plasmid pSXC4-2 carrying IncFIN/IncHI1B/IncR/IncX1 types from *E. coli* strains isolated from chicken sources in Jiangsu, and the plasmid pHNMCC14 carrying IncFII/IncN/IncN/IncX1 types from chicken-derived *E. coli* strains in Guangzhou. Additionally, it is worth noting that elements related to conjugative transfer such as *oriT*, T4SS, T4CP and relaxase were not found in the plasmid pfosA3-LA3. Therefore, this may be a reason for the inability of *fos*A3-positive plasmids in strain LA3 to undergo conjugative transfer.

The dissemination of ARGs is not confined to plasmids, it also includes other mobile genetic structures, such as transposons and insertion elements. IS*26* plays a crucial role in the spread and mobilization of *fos*A3 (63). It can facilitate the transfer of ARGs by forming a circular intermediate structure, thereby promoting their dissemination through replicative transposition (71). In this study, it was found that there is an insertion element IS*26* both upstream and downstream of the *fos*A3 in three *fos*A3-positive plasmids, forming a specific genetic structure of IS*26-hp-hp-hp-fos*A3-IS*26*. This is a common genetic structure for *fos*A3, which has been reported in multiple studies (9). Previous research has shown that *fos*A3 often co-disseminates with *bla*_CTX-M_ (7, 55). In the plasmids p*fos*A3-BHD6 and p*fos*A3-HN68, it was found that both *bla*_TEM_ and *bla*_CTX-M_ are present upstream of *fos*A3. Additionally, there is an IS*26* sequence upstream of *bla*_CTX-M_, along with a truncated 131 bp IS*1* family mobile element. Six genetic structures of *fos*A3 were identified in animal-derived *E. coli*, with three core structures similar to those in this study. Additionally, three unique genetic structures were found: IS*26*-CTX-M-15-IS*5*/IS*1182*-*fos*A3-*orf*1-*orf*2-*orf*3-IS*26*; IS*26-fos*A3-*orf1*-IS*26*; and IS*26*-CTX-M-55-TEM-1-IS*26*-*fos*A3-*orf1*-IS*26* (10). Two new genetic environments of *fos*A3 were first discovered in ESBL-producing *E. coli* from chicken meat in China (IS*Ecp1*-*bla*_CTX-M-65_-ΔIS*903D*-IS*26*-*fos*A3-*orf1*-*orf2*-Δ*orf3*-IS*26* and IS*26*- IS*Ecp1*-*bla*_CTX-M-3_-*orf477*-*bla*_TEM-1_-IS*26*-*fos*A3-*orf1*-*orf2*-Δ*orf3*-IS*26*) (32). These findings provide critical insights into understanding of the transmission mechanism and genetic background of *fos*A3.

## Conclusion

Our findings revealed that FOS resistance *E. coli* in waterfowl filed in selected regions is mainly due to the plasmid-mediated *fos*A3. The presence of both *fos*A3 and *bla*_CTX-M_ may have contributed to the rapid spread of MDR bacteria. The spread of *fos*A3 is primarily attributed to the horizontal transfer of plasmids with high conjugation ability, along with clonal dissemination. It is significant to note that the *fos*A3 is often flanked by IS*26*, which could be crucial for its transfer between plasmids. Considering the ecological role of waterfowl and the clinical importance of FOS, the emergence of FOS resistance could potentially spread through the food chain and the environment, posing a risk to public health. Therefore, reducing the use of related antibiotics in Chinese poultry farming may be effective in curbing the spread of the *fos*A3 in *E. coli.*

## Data Availability

The datasets presented in this study can be found in online repositories. The names of the repository/repositories and accession number(s) can be found in the article/Supplementary material.

## Footnotes

^1^ http://www.ncbi.nlmnih.gov/BLAST/

^2^ https://pubmlst.org/

^3^ https://www.genomicepidemiology.org/

^4^ https://cgview.ca/
