# Supplementary material for: High prevalence of plasmid-mediated Fosfomycin resistance in waterfowl-derived Escherichia coli strains: insights into genetic context and transmission dynamics in China
Source: Front Vet Sci. 2025 Mar 21;12:1481822. doi: 10.3389/fvets.2025.1481822 (PMC11969801; doi:10.3389/fvets.2025.1481822)
Supplement: Supplementary file 1 [file Table_1.docx]

**Table S1 Primer sequences for ARGs**

| Primers | primer sequence(5’-3’) | Product size(bp) | references |
| --- | --- | --- | --- |
| *fos*A-F | ATCTGTGGGTCTGCCTGTCGT | 271 | (1) |
| *fos*A-R | ATGCCCGCATAGGGCTTCT |  |  |
| *fos*A2-F | GCAATCACTCAACCATCTGACC | 404 | (2) |
| *fos*A2-R | TGAAGACCATTCCGGCATAGG |  |  |
| *fos*A3-F | GCGTCAAGCCTGGCATTT | 282 | (1) |
| *fos*A3-R | GCCGTCAGGGTCGAGAAA |  |  |
| *fos*A4-F | CTGGCGTTTTATCAGCGGTT | 230 | (3) |
| *fos*A4-R | CTTCGCTGCGGTTGTCTTT |  |  |
| *fos*A5-F | TATTAGCGAAGCCGATTTTGCT | 177 | (3) |
| *fos*A5-R | CCCCTTATACGGCTGCTCG |  |  |
| *fos*A6-F | CGAGCGTGGCGTTTTATCAG | 194 | (3) |
| *fos*A6-R | GGCGAAGCTAGCAAAATCGG |  |  |
| *fos*A7-F | CTCTGAACCACTTAACGCT | 270 | (4) |
| *fos*A7-R | CTTTCCAGACCGTCACTCC |  |  |
| *fos*A8-F | AACCATCTGACCCTTGCTGT | 301 | (5) |
| *fos*A8-R | CGAGAAAATAGAACGACGCC |  |  |
| *fos*A9-F | CACCTGACCCTGGCAGTCAG | 326 | (6) |
| *fos*A9-R | GGCCATAAGCTGGAGCTGCA |  |  |
| *fos*A10-F | CGAGCGTGGCGTTTTATCAG | 278 | (4) |
| *fos*A10-R | GCCATCGGGATCGAGGAAAT |  |  |
| *tet*A-F | GTAATTCTGAGCACTGTCGC | 937 | (7) |
| *tet*A-R | CTGCCTGGACAACATTGCTT |  |  |
| *tet*B-F | CTCAGTATTCCAAGCCTTTG | 416 | (7) |
| *tet*B-R | CTAAGCACTTGTCTCCTGTT |  |  |
| *tet*C-F | TCTAACAATGCGCTCATCGT | 570 | (7) |
| *tet*C-R | GGTTGAAGGCTCTCAAGGGC |  |  |
| *tet*O-F | AACTTAGGCATTCTGGCTCAC | 515 | (8) |
| *tet*O-R | TCCCACTGTTCCATATCGTCA |  |  |
| *tet*M-F | GTGGACAAAGGTACAACGAG | 406 | (8) |
| *tet*M-R | CGGTAAAGTTCGTCACACAC |  |  |
| *sul1-*F | GTGACGGTGTTCGGCATTCT | 779 | (9) |
| *sul1*-R | CCGAGAAGGTGATTGCGCT |  |  |
| *sul2*-F | CGGCATCGTCAACATAACC | 722 | (10) |
| *sul2-*R | GTGTGCGGATGAAGTCAG |  |  |
| *sul3* -F | CATTCTAGAAAACAGTCGTAGTCG | 990 | (11) |
| *sul3* -R | CATCTGCAGCTAACTAGGGCTTTGGA |  |  |
| *mcr-1*-F | AGTCCGTTTGTTCTTGTGGC | 320 | (12) |
| *mcr-1*-R | AGATCCTTGGTCTCGGCTTG |  |  |
| *cml*A-F | TGTCATTTACGGCATACTCG | 455 | (13) |
| *cml*A-R | ATCAGGCATCCCATTCCCAT |  |  |
| *floR*-F | GTCGAGAAATCCCATGAGTTCA | 1645 | (14) |
| *floR*-R | CAGACAGGATACCGACATTCAC |  |  |
| *fexA*-F | GTACTTGTAGGTGCAATTACGGCTGA | 1272 | (15) |
| *fexA*-R | CGCATCTGAGTAGGACATAGC GTC |  |  |
| *bla*_TEM_-F | GCGGAACCCCTATTTG | 964 | (16) |
| *bla*_TEM_-R | ACCAATGCTTAATCAGTGAG |  |  |
| *bla*_SHV_-F | TTATCTCCCTGTTAGCCACC | 795 | (16) |
| *bla*_SHV_-R | GATTTGCTGATTTCGCTCGG |  |  |
| *bla*_CTX-M_-F | CGATGTGCAGTACCAGTAA | 585 | (16) |
| *bla*_CTX-M_-R | TAGTGACCAGAATCAGCGG |  |  |
| *bla*_NDM_-F | ATGGAATTGCCCAATATTATGCAC | 813 | (16) |
| *bla*_NDM_*-R* | TCAGCGCAGCTTGTCGGC |  |  |
| *qnrA-*F | ATTTCTCACGCCAGGATTTG | 516 | (17) |
| *qnrA-*R | GATCGGCAAAGGTTAGGTCA |  |  |
| *qnrB*-F | GATCGTGAAAGCCAGAAAGG | 469 | (17) |
| *qnrB*-R | ACGATGCCTGGTAGTTGTCC |  |  |
| *qnrS*-F | ACGACATTCGTCAACTGCAA | 417 | (17) |
| *qnrS-*R | TAAATTGGCACCCTGTAGGC |  |  |
| *rmt*A-F | CTAGCGTCCATCCTTTCCTC | 635 | (18) |
| *rmt*A-R | TTGCTTCCATGCCCTTGCC |  |  |
| *rmt*B-F | GCTTTCTGCGGGCGATGTAA | 173 | (18) |
| *rmt*B-R | ATGCAATGCCGCGCTCGTAT |  |  |
| *aph*A1-F | ATGGGCTCGCGATAATGTC | 600 | (10) |
| *aph*A1-R | CTCACCGAGGCAGTTCCAT |  |  |
| *aad*A1*-*F | TATCAGAGGTAGTTGGCGTCAT | 489 | (19) |
| *aad*A1*-*R | GCGAGTTCCATAGCGTTAAGG |  |  |
| *aac(6’)-Ib-cr*-F | TTGCGATGCTCTATGAGTGGCTA | 482 | (20) |
| *aac(6’)-Ib-cr*-R | CTCGAATGCCTGGCGTGTTT |  |  |
| *aac(3’)*-III-F | CACAAGAACGTGGTCCGCTA | 185 | (13) |
| *aac(3’)*-III-R | AACAGGTAAGCATCCGCATC |  |  |
| *aac(3’)*-IV-F | CTTCAGGATGGCAAGTTGGT | 286 | (13) |
| *aac(3’)*-IV-R | TCATCTCGTTCTCCGCTCAT |  | (21) |
| I2-F | CTGTCGGCATGTCTGTCTC | 552 |  |
| I2-R | CTGGCTACCAGTTGCTCTAA |  |  |
| HI1-F | GGAGCGATGGATTACTTCAGTAC | 471 |  |
| HI1-R | TGCCGTTTCACCTCGTGAGTA |  |  |
| HI2-F | TTTCTCCTGAGTCACCTGTTAACAC | 644 |  |
| HI2-R | GGCTCACTACCGTTGTCATCCT |  |  |
| I1-F | CGAAAGCCGGACGGCAGAA | 139 | (21) |
| I1-R | TCGTCGTTCCGCCAAGTTCGT |  |  |
| X-F | AACCTTAGAGGCTATTTAAGTTGCTGAT | 376 |  |
| X-R | TGAGAGTCAATTTTTATCTCATGTTTTAGC |  |  |
| L/M-F | GGATGAAAACTATCAGCATCTGAAG | 785 |  |
| L/M-R | CTGCAGGGGCGATTCTTTAGG |  |  |
| N-F | GTCTAACGAGCTTACCGAAG | 559 |  |
| N-R | GTTTCAACTCTGCCAAGTTC |  |  |
| FIA-F | CCATGCTGGTTCTAGAGAAGGTG | 462 |  |
| FIA-R | GTATATCCTTACTGGCTTCCGCAG |  |  |
| FIB-F | GGAGTTCTGACACACGATTTTCTG | 702 |  |
| FIB-R | CTCCCGTCGCTTCAGGGCATT |  |  |
| W-F | CCTAAGAACAACAAAGCCCCCG | 242 |  |
| W-R | GGTGCGCGGCATAGAACCGT |  |  |
| Y-F | AATTCAAACAACACTGTGCAGCCTG | 765 |  |
| Y-R | GCGAGAATGGACGATTACAAAACTTT |  |  |
| P-F | CTATGGCCCTGCAAACGCGCCAGAAA | 534 |  |
| P-R | TCACGCGCCAGGGCGCAGCC |  |  |
| FIC-F | GTGAACTGGCAGATGAGGAAGG | 262 |  |
| FIC-R | TTCTCCTCGTCGCCAAACTAGAT |  |  |
| A/C-F | GAGAACCAAAGACAAAGACC TGGA | 465 |  |
| A/C-R | ACGACAAACCTGAATTGCCTCCTT |  |  |
| T-F | TTGGCCTGTTTGTGCCTAAACCAT | 750 |  |
| T-R | CGTTGATTACACTTAGCTTTGGAC |  |  |
| FIIS-F | CTGTCGTAAGCTGATGGC | 270 |  |
| FIIS-R | CTCTGCCACAAACTTCAGC |  |  |
| FrepB-F | TGATCGTTTAAGGAATTTTG | 270 |  |
| FrepB-R | GAAGATCAGTCACACCATCC |  |  |
| K/B-F | GCGGTCCGGAAAGCCAGAAAAC | 160 |  |
| K-R | TCTTTCACGAGCCCGCCAAA |  |  |
| B/O-R | TCTGCGTTCCGCCAAGTTCGA | 159 |  |

**References:**

1. Hou J, Huang X, Deng Y, He L. Dissemination of the fosfomycin resistance gene fosA3 with CTX-M β-lactamase genes and rmtB carried on IncFII plasmids among escherichia coli isolates from pets in china. *Antimicrob Agents Chemother* (2012) 56(4):2135–8:

2. Xu H, Miao V, Kwong W, Xia R, Davies J. Identification of a novel fosfomycin resistance gene (fosA2) in enterobacter cloacae from the salmon river, canada. *Lett Appl Microbiol* (2011) 52:427–429. doi: 10.1111/j.1472-765X.2011.03016.x

3. Loras C, González-Prieto A, Pérez-Vázquez M, Bautista V, Ávila A, Campoy PS, Oteo-Iglesias J, Alós J-I. Prevalence, detection and characterisation of fosfomycin-resistant escherichia coli strains carrying fosA genes in community of madrid, spain. *J Glob Antimicrob Resist* (2021) 25:137–141. doi: 10.1016/j.jgar.2021.02.032

4. Zhang X, Ma M, Cheng Y, Huang Y, Tan Y, Yang Y, Qian Y, Zhong X, Lu Y, Si H. Spread and molecular characteristics of enterobacteriaceae carrying fosA-like genes from farms in china. *Microbiol Spectr* (2022) 10:e0054522. doi: 10.1128/spectrum.00545-22

5. Poirel L, Vuillemin X, Kieffer N, Mueller L, Descombes M-C, Nordmann P. Identification of FosA8, a plasmid-encoded fosfomycin resistance determinant from escherichia coli, and its origin in leclercia adecarboxylata. *Antimicrob Agents Chemother* (2019) 63:e01403-19. doi: 10.1128/AAC.01403-19

6. Freire S, Grilo T, Nordmann P, Poirel L, Aires-de-Sousa M. Multiplex PCR for detection of acquired plasmid-borne fosfomycin resistance fos genes in escherichia coli. *Diagn Microbiol Infect Dis* (2023) 105:115864. doi: 10.1016/j.diagmicrobio.2022.115864

7. Guardabassi L, Dijkshoorn L, Collard J-M, Olsen JE, Dalsgaard A. Distribution and in-vitro transfer of tetracycline resistance determinants in clinical and aquatic acinetobacter strains. *J Med Microbiol* (2000) 49:929–936. doi: 10.1099/0022-1317-49-10-929

8. Gholami-Ahangaran M, Karimi-Dehkordi M, Miranzadeh-Mahabadi E, Ahmadi-Dastgerdi A. The frequency of tetracycline resistance genes in escherichia coli strains isolated from healthy and diarrheic pet birds. *Iran J Vet Res* (2021) 22:337–341. doi: 10.22099/ijvr.2021.38454.5592

9. Boerlin P, Travis R, Gyles CL, Reid-Smith R, Janecko N, Lim H, Nicholson V, McEwen SA, Friendship R, Archambault M. Antimicrobial resistance and virulence genes of escherichia coli isolates from swine in ontario. *Appl Environ Microbiol* (2005) 71:6753–6761. doi: 10.1128/AEM.71.11.6753-6761.2005

10. Maynard C, Fairbrother JM, Bekal S, Sanschagrin F, Levesque RC, Brousseau R, Masson L, Larivière S, Harel J. Antimicrobial resistance genes in enterotoxigenic escherichia coli o149:k91 isolates obtained over a 23-year period from pigs. *Antimicrob Agents Chemother* (2003) 47:3214–3221. doi: 10.1128/AAC.47.10.3214-3221.2003

11. Ramos S, Silva N, Caniça M, Capelo-Martinez JL, Brito F, Igrejas G, Poeta P. High prevalence of antimicrobial-resistant escherichia coli from animals at slaughter: a food safety risk. *J Sci Food Agric* (2013) 93:517–526. doi: 10.1002/jsfa.5814

12. Rebelo AR, Bortolaia V, Kjeldgaard JS, Pedersen SK, Leekitcharoenphon P, Hansen IM, Guerra B, Malorny B, Borowiak M, Hammerl JA, et al. Multiplex PCR for detection of plasmid-mediated colistin resistance determinants, mcr-1, mcr-2, mcr-3, mcr-4 and mcr-5 for surveillance purposes. *Eurosurveillance* (2018) 23:17–00672. doi: 10.2807/1560-7917.ES.2018.23.6.17-00672

13. Sáenz Y, Briñas L, Domínguez E, Ruiz J, Zarazaga M, Vila J, Torres C. Mechanisms of resistance in multiple-antibiotic-resistant escherichia coli strains of human, animal, and food origins. *Antimicrob Agents Chemother* (2004) 48:3996–4001. doi: 10.1128/AAC.48.10.3996-4001.2004

14. Cloeckaert A, Baucheron S, Flaujac G, Schwarz S, Kehrenberg C, Martel JL, Chaslus-Dancla E. Plasmid-mediated florfenicol resistance encoded by the floR gene in escherichia coli isolated from cattle. *Antimicrob Agents Chemother* (2000) 44:2858–2860. doi: 10.1128/AAC.44.10.2858-2860.2000

15. Zhang A, Yang Y, Wang H, Lei C, Xu C, Guan Z, Liu B, Huang X, Peng L. Prevalence of sulfonamide and florfenicol resistance genes in escherichia coli isolated from yaks (bos grunniens) and herdsmen in the tibetan pasture. *J Wildl Dis* (2015) 51:626–633. doi: 10.7589/2014-09-234

16. Yang H, Rehman MU, Zhang S, Yang J, Li Y, Gao J, Gu L, Wang M, Jia R, Chen S, et al. High prevalence of CTX-M belonging to ST410 and ST889 among ESBL producing E. coli isolates from waterfowl birds in china’s tropical island, hainan. *Acta Trop* (2019) 194:30–35. doi: 10.1016/j.actatropica.2019.03.008

17. Robicsek A, Strahilevitz J, Sahm F, 雅各比 G, 胡珀哥伦比亚特区. Qnr prevalence in ceftazidime-resistantenterobacteriaceae isolates from the united states. *Antimicrob Agents Chemother* (2006) 50:2872–2874. doi: 10.1128/aac.01647-05

18. Wang Y, Zhang A, Yang Y, Lei C, Jiang W, Liu B, Shi H, Kong L, Cheng G, Zhang X, et al. Emergence of salmonella enterica serovar indiana and california isolates with concurrent resistance to cefotaxime, amikacin and ciprofloxacin from chickens in china. *Int J Food Microbiol* (2017) 262:23–30. doi: 10.1016/j.ijfoodmicro.2017.09.012

19. Niu J-L, Peng J-J, Ming Y-Y, Ma Q-C, Liu W-C, Ma Y. Identification of drug resistance genes and drug resistance analysis of salmonella in the duck farm environment of zhanjiang, china. *Environ Sci Pollut Res Int* (2020) 27:24999–25008. doi: 10.1007/s11356-020-09007-5

20. Eftekhar F, Seyedpour SM. Prevalence of qnr and aac(6’)-ib-cr genes in clinical isolates of klebsiella pneumoniae from imam hussein hospital in tehran. *Iran J Med Sci* (2015) 40:515–521.

21. Zhang S, Yang H, Rehman MU, Yang K, Dong M, Yang J, et al. Class 1 integrons as predominant carriers in *Escherichia* *coli* isolates from waterfowls in Hainan, China. *Ecotoxicol* Environ Saf. (2019) 183:109514. doi: 10.1016/j.ecoenv.2019.109514
