# Supplementary material for: High prevalence of plasmid-mediated Fosfomycin resistance in waterfowl-derived Escherichia coli strains: insights into genetic context and transmission dynamics in China
Source: Front Vet Sci. 2025 Mar 21;12:1481822. doi: 10.3389/fvets.2025.1481822 (PMC11969801; doi:10.3389/fvets.2025.1481822)
Supplement: Supplementary file 2 [file Table_3.docx]

**Table S3 Odds ratios (OR) of ARGs in fosfomycin resistant *E.* *coli***

| **gene** | ***tet*A(79)** | ***aph*A1(69)** | ***sul*2(65)** | ***flo*R (62)** | ***qnrS(*58)** | ***bla*_CTX-M_(52)** | ***aad*A1(36)** | ***cml*A(34)** | ***sul*1 (26)** | ***aac(6')-ib-cr*(25)** | ***tet*B(16)** | ***qnr*A(14)** | ***aac(3')-III(8)*** |
| --- | --- | --- | --- | --- | --- | --- | --- | --- | --- | --- | --- | --- | --- |
| ***Tet*A(79)** | **NS** | **4.211 (1.539-11.521)** | **-** | **-** | **9.180 (2.799-30.112)** | **-** | **0.253 (0.092-0.691)** | **-** | **-** | **-** | **-** | **-** | ***-*** |
| ***aph*A1(69)** | **4.211**  **(1.539-11.521)** | **NS** | **-** | **-** | **3.167 (1.315-7.628)** | **-** | **0.313 (0.129-0.757)** | **-** | **-** | **4.369 (1.198-15.932)** | **-** | **6.964 (0.869-55.842)** | ***-*** |
| ***sul*2(65)** | **-** | **-** | **NS** | **-** | **-** | **-** | **-** | **0.455 (0.193-1.0873)** | **-** | **-** | **4.529**  **(0.966-21.233)** | **-** | ***-*** |
| ***flo*R(62)** | **-** | **-** | **-** | **NS** | **-** | **-** | **-** | **-** | **-** | **-** | **-** | **-** | ***-*** |
| ***qnr*S(58)** | **9.180**  **(2.799-30.112)** | **3.167(1.315-7.628)** | **-** | **-** | **NS** | **-** | **-** | **-** | **-** | **-** | **-** | **5.217 (1.101-24.724)** | ***-*** |
| ***bla*_CTX-M_ (52)** | **-** | **-** | **-** | **-** | **-** | **NS** | **-** | **-** | **-** | **-** | **3.300**  **(0.984-11.065)** | **-** | ***-*** |
| ***aad*A1(36)** | **0.253**  **(0.092-0.691)** | **0.313 (0.129-0.757)** | **-** | **-** | **-** | **-** | **NS** | **-** | **-** | **-** | **-** | **-** | ***-*** |
| ***cml*A(34)** | **-** | **-** | **0.455 (0,193-1.0873)** | **-** | **-** | **-** | **-** | **NS** | **-** | **-** | **-** | **-** | ***-*** |
| ***sul*1(26)** | **-** | **-** | **-** | **-** | **-** | **-** | **-** | **-** | **NS** | **-** | **-** | **-** | ***-*** |
| ***aac(6')-ib-cr(25)*** | **-** | **4.369 (1.198-15.932)** | **-** | **-** | **-** | **-** | **-** | **-** | **-** | **NS** | **-** | **-** | ***-*** |
| ***tet*B(16)** | **-** | **-** | **4.529 (0.966-21.233)** | **-** | **-** | **3.300(0.984-11.065)** | **-** | **-** | **-** | **-** | **NS** | **-** | **6.667 (1.468-30.266)** |
| ***qnr*A(14)** | **-** | **6.964**  **(0.869-55.842)** | **-** | **-** | **5.217 (1.101-24.724)** | **-** | **-** | **-** | **-** | **-** | **-** | **NS** | **-** |
| ***aac(3')-*III(8)** | **-** | **-** | **-** | **-** | **-** | **-** | **-** | **-** | **-** | **-** | **6.667 (1.468-30.266)** | **-** | **NS** |
| ***bla*_SHV_ (2)** | **-** | **-** | **-** | **-** | **-** | **-** | **-** | **1.063 (0.977-1.156)** | **-** | **-** | **-** | **-** | **-** |
| ***mcr*-1(1)** | **-** | **-** | **-** | **-** | **-** | **-** | **-** | **-** | **-** | **-** | **-** | **-** | **-** |
| ***sul*3(1)** | **-** | **-** | **-** | **-** | **-** | **-** | **-** | **-** | **-** | **-** | **-** | **-** | **-** |
| ***tet*C(1)** | **-** | **-** | **-** | **-** | **-** | **-** | **-** | **-** | **-** | **-** | **-** | **-** | **-** |
| ***rtm*B(18)** | **-** | **0.205**  **(0.070-0.600)** | **-** | **-** | **0.210**  **(0.068-0.649)** | **-** | **3.583(1.244-10.319)** | **-** | **-** | **-** | **-** | **-** | **-** |
| ***bla*_NDM-5_(1)** | **-** | **-** | **-** | **-** | **-** | **-** | **-** | **-** | **-** | **-** | **-** | **-** | **-** |
| ***qnr*B(1)** | **-** | **-** | **-** | **-** | **-** | **-** | **-** | **-** | **-** | **-** | **-** | **-** | **-** |
| ***bla*_TEM_(56)** | **-** | **-** | **-** | **-** | **-** | **0.429**  **(0.191-0.94)** | **-** | **-** | **-** | **-** | **-** | **0.099 (0.21-0.470)** | **-** |
| ***fos*A(11)** | **0.063**  **(0.015-0.274)** | **0.211 (0.057-0.0786)** | **-** | **-** | **0.056 (0.007-0.459)** | **0.75**  **(0.009-0.607)** | **-** | **-** | **-** | **-** | **-** | **-** | **-** |
| ***fos*A3(88)** | **7.4**  **(2.053-.26.667)** | **-** | **-** | **-** | **5.0**  **(1.263-19.797)** | **15.162 (1.875-122.635)** | **-** | **-** | **-** | **-** | **-** | **-** | **-** |
| ***fos*A6(2)** | **-** | **-** | **-** | **-** | **-** | **-** | **-** | **-** | **-** | **-** | **-** | **-** | **-** |
| ***fos*A7(3)** | **-** | **-** | **-** | **-** | **-** | **-** | **-** | **-** | **-** | **-** | **-** | **-** | **-** |
| ***fos*A10(3)** | **-** | **-** | **-** | **-** | **-** | **-** | **-** | **-** | **-** | **-** | **-** | **-** | **-** |

**Table S3 Odds ratios (OR) of ARGs in fosfomycin *E.* *coli* (continuation sheet)**

| **gene** | ***bla*_SHV_(2)** | ***mcr*-1(1)** | ***sul*3(1)** | ***tet*C(1)** | ***rtm*B(18)** | ***bla*_NDM-5_(1)** | ***qnr*B(1)** | ***bla*_TEM_(56)** | ***fos*A(11)** | ***fos*A3(88)** | ***fos*A6(2)** | ***fos*A7(3)** | ***fos*A10(3)** |
| --- | --- | --- | --- | --- | --- | --- | --- | --- | --- | --- | --- | --- | --- |
| ***tet*A(79)** | - | - | - | - | **-** | **-** | **-** | **-** | **0.063(0.015-0.274)** | **7.4(2.053-.26.667)** | **-** | **-** | **-** |
| ***aph*A1(69)** | - | - | - | - | **0.205(0.070-0.600)** | **-** | **-** | **-** | **0.211(0.057-0.0786)** | **-** | **-** | **-** | **-** |
| ***sul*2(65)** | - | - | - | - | **-** | **-** | **-** | **-** | **-** | **-** | **-** | **-** | **-** |
| ***flo*R(62)** | - | - | - | - | **-** | **-** | **-** | **-** | **-** | **-** | **-** | **-** | **-** |
| ***qnr*S(58)** | - | - | - | - | **0.210(0.068-0.649)** | **-** | **-** | **-** | **0.056(0.007-0.459)** | **5.0(1.263-19.797)** | **-** | **-** | **-** |
| ***bla*_CTX-M_(52)** | - | - | - | - | **-** | **-** | **-** | **0.429(0.191-0.94)** | **0.75(0.009-0.607)** | **15.162(1.875-122.635)** | **-** | **-** | **-** |
| ***aad*A1(36)** | - | - | - | - | **3.583(1.244-10.319)** | **-** | **-** | **-** | **-** | **-** | **-** | **-** | **-** |
| ***cml*A(34)** | - | - | - | - | **-** | **-** | **-** | **-** | **-** | **-** | **-** | **-** | **-** |
| ***sul*1(26)** | - | - | - | - | **-** | **-** | **-** | **-** | **-** | **-** | **-** | **-** | **-** |
| ***aac(6')-ib-cr*(25)** | - | - | - | - | **-** | **-** | **-** | **-** | **-** | **-** | **-** | **-** | **-** |
| ***tet*B(16)** | - | - | - | - | **-** | **-** | **-** | **-** | **-** | **-** | **-** | **-** | **-** |
| ***qnr*A(14)** | - | - | - | - | **-** | **-** | **-** | **0.099(0.21-0.470)** | **-** | **-** | **-** | **-** | **-** |
| ***aac*(3')-III(8)** | - | - | - | - | **-** | **-** | **-** | **-** | **-** | **-** | **-** | **-** | **-** |
| ***bla*_SHV_ (2)** | **NS** | **-** | **-** | **-** | **-** | **-** | **-** | **-** | **-** | **-** | **-** | **-** | **-** |
| ***mcr*-1(1)** | **-** | **NS** | **-** | **-** | **-** | **-** | **-** | **-** | **-** | **-** | **-** | **-** | **-** |
| ***sul*3(1)** | **-** | **-** | **NS** | **-** | **-** | **-** | **-** | **-** | **-** | **-** | **-** | **-** | **-** |
| ***tet*C(1)** | **-** | **-** | **-** | **NS** | **-** | **-** | **-** | **-** | **-** | **-** | **-** | **-** | **-** |
| ***rtm*B(18)** | **-** | **-** | **-** | **-** | **NS** | **-** | **-** | **3.333(1.011-10.985)** | **12.409(3.118-49.382)** | **0.102(0.028-0.378)** | **-** | **-** | **-** |
| ***bla*_NDM-5_(1)** | **-** | **-** | **-** | **-** | **-** | **NS** | **-** | **-** | **-** | **-** | **-** | **-** | **-** |
| ***qnr*B(1)** | **-** | **-** | **-** | **-** | **-** | **-** | **NS** | **-** | **-** | **-** | **-** | **-** | **-** |
| ***bla*_TEM_(56)** | **-** | **-** | **-** | **-** | **3.333(1.011-10.985)** | **-** | **-** | **NS** | **-** | **-** | **-** | **-** | **-** |
| ***fos*A(11)** | **-** | **-** | **-** | **-** | **12.409(3.118-49.382)** | **-** | **-** | **-** | **NS** | **0.008(0.001-0.053)** | **-** | **-** | **-** |
| ***fos*A3(88)** | **-** | **-** | **-** | **-** | **0.102(0.028-0.378)** | **-** | **-** | **-** | **0.008(0.001-0.053)** | **NS** | **-** | **-** | **-** |
| ***fos*A6(2)** | **-** | **-** | **-** | **-** | **-** | **-** | **-** | **-** | **-** | **-** | **NS** | **-** | **-** |
| ***fos*A7(3)** | **-** | **-** | **-** | **-** | **-** | **-** | **-** | **-** | **-** | **-** | **-** | **NS** | **-** |
| ***fos*A10(3)** | **-** | **-** | **-** | **-** | **-** | **-** | **-** | **-** | **-** | **-** | **-** | **-** | **NS** |

Note: Only ARGs with significant correlations (*p* < 0.05) are shown in the table. OR indicates the ratio of significant associations between the corresponding ARGs (95% confidence intervals in parentheses); NS indicates that statistical analyses could not be performed on the same genes; and "-" indicates that the results were not available (or) could not be calculated
